# CAR signaling instructs divergent metabolic reprogramming and functional fates in αβ and γδ T cells

**DOI:** 10.1038/s41423-026-01475-y

**Published:** 2026-09-30

**Authors:** Xiomar E. Bustos, Leticia Tordesillas, Elena Martinez Planes, Miguel G. Fontela, Sebastian Snedal, Renata Ariza Marques Rossetti, Victoria Izumi, Bin Fang, John M. Koomen, Eric A. Welsh, Patrick Hwu, Daniel Abate-Daga

**Affiliations:** 1 Department of Immunology, H. Lee Moffitt Cancer Center & Research Institute, Tampa, FL USA; 2 Proteomics & Metabolomics Core, H. Lee Moffitt Cancer Center & Research Institute, Tampa, FL USA; 3 Molecular Oncology, H. Lee Moffitt Cancer Center & Research Institute, Tampa, FL USA; 4 Department of Biostatistics and Bioinformatics, H. Lee Moffitt Cancer Center & Research Institute, Tampa, FL USA; 5 Department of Cutaneous Oncology, H. Lee Moffitt Cancer Center & Research Institute, Tampa, FL USA

**Keywords:** AP-1 transcription factor, c-Jun, PSCA CAR-T cell, RANKL, TXNIP, Translational immunology, Cancer immunotherapy

## Abstract

γδ T-cell-based immunotherapies have become relevant as alternatives to conventional αβ T-cell products, with preclinical data demonstrating tumor burden reduction and the mitigation of tumor-induced tissue damage. Given that most CAR constructs have been optimized for αβ T cells, we hypothesized that distinct T-cell types may require tailored CAR architectures to achieve optimal function. To test this hypothesis, we conducted a systematic comparative analysis of γδ and αβ T cells transduced with a second-generation PSCA-targeting CAR (PSCA-8t28z). We found that although γδ and αβ CAR-T cells exhibit comparable levels of cytotoxicity, they differ phenotypically. Through a system-level phosphoproteomic analysis, we identified 307 phosphosites whose abundance differed between γδ and αβ CAR-T cells. Pathway enrichment analysis placed glycolysis/gluconeogenesis and TCR signaling within the top significantly overrepresented signaling networks. The results of functional validation studies confirmed that γδ CAR-T cells have lower glycolytic and oxidative phosphorylation capacity than αβ-CAR-T cells do and weaker activation of activator protein 1 (AP-1). Notably, we identified thioredoxin-interacting protein (TXNIP) as a potential actionable target to enhance γδ CAR-T-cell metabolism. Finally, we designed a new synthetic costimulatory receptor that potentiates AP-1 activation, resulting in improved in vivo persistence. These results highlight the fundamental biological differences between γδ and αβ T cells and support the development of cell type-specific receptor engineering strategies to maximize γδ CAR-T-cell function and therapeutic benefit.

## Introduction

Gamma delta (γδ) T-cell-based immunotherapies represent an emerging class of adoptive cell therapies (ACTs) with the potential to overcome several limitations associated with traditional alpha beta (αβ) T-cell products [1–4]. Unlike αβ T cells, γδ T cells exhibit innate-like cytotoxicity and recognize stress-induced or nonpeptide antigens in a manner that is independent of classical MHC restriction [5, 6]. In addition to their direct tumor-killing ability, γδ T cells have demonstrated the capacity for antigen cross-presentation [7, 8], further amplifying immune responses. These characteristics render them especially attractive for use in both autologous and allogeneic contexts [5, 9–11]. Among γδ T-cell subsets, Vδ2 and Vδ1 T cells preferentially home to inflamed or malignant tissues and select nonmalignant niches, making them particularly relevant for targeting solid tumors [12, 13].

Clinical trials of unmodified γδ T cells have consistently demonstrated a favorable safety profile [14–17]. However, clinical responses have been modest, underscoring the need for strategies that enhance their effector function and persistence. Genetic modification, most notably through the expression of chimeric antigen receptors (CARs), has emerged as a promising avenue to unlock the full therapeutic potential of γδ T cells. Indeed, γδ CAR-T cells have shown encouraging preclinical activity against a range of solid tumors, including hepatocellular carcinoma [18], ovarian [19], gastric [20], and metastatic prostate cancer [21]. Beyond CAR expression, ongoing efforts aim to optimize the intrinsic features of γδ T cells, including their cytotoxicity, cytokine profile, and metabolic resilience [22].

To date, most CAR design efforts have been guided by studies in αβ T cells, which are better characterized and more readily obtained from peripheral blood [23]. CAR architecture, which includes costimulatory domains, hinges, and transmembrane sequences, has a profound effect on T-cell phenotype and function, influencing cytotoxicity, persistence, memory differentiation, cytokine release, and cellular metabolism [24–27]. CAR-T cells are expected to function in nutrient-deprived, hypoxic tumor microenvironments. Because of this limiting environment, improving metabolic performance has become a key objective [28–30]. Approaches to enhancing glycolytic capacity and oxidative phosphorylation have yielded promising results in αβ CAR-T cells [31–33], yet many of these metabolic strategies remain untested in γδ T cells.

Despite the growing interest in γδ T-cell-based therapies, the fundamental differences in their biology remain poorly understood [34, 35]. Consequently, γδ CAR-T-cell engineering has relied heavily on αβ-derived design principles under the assumption of functional homology between these lineages. However, whether these αβ-based CAR constructs are optimal for γδ T cells is unclear. In this context, we sought to elucidate the signaling and functional consequences of CAR engagement in γδ T cells versus αβ T cells to inform the rational design of gene modifications tailored to γδ T cells.

In this study, we provide a comparative analysis of CAR-triggered signaling in αβ and γδ T cells, particularly Vγ9Vδ2, with a focus on effector function and metabolic programming. Our results demonstrate that CAR signaling in γδ T cells leads to distinct pathways and divergent activation profiles. Notably, we detected significant differences in the metabolic phenotype between Vγ9Vδ2 T cells, which exhibit lower glycolytic activity and lower mitochondrial activity, and αβ T cells. We observed an improvement in mitochondrial respiration activity in expanded Vγ9Vδ2 T cells following inhibition of thioredoxin-interacting protein (TXNIP), which is highly expressed in Vγ9Vδ2 T cells. TXNIP inhibition during T-cell expansion resulted in improved spare respiratory capacity, mitochondrial mass, and CAR expression, suggesting a feasible strategy to increase γδ T-cell metabolic fitness.

In parallel, we identified differential engagement of canonical TCR signaling components downstream of CAR activation, including diminished activation of key transcription factors, e.g., AP-1 and nuclear factor of activated T cells (NFAT), in γδ CAR-T cells. To increase AP-1 activation, we designed a novel chimeric switch receptor (CSR) that leverages the natural abundance of receptor activator of nuclear factor κ B-ligand (RANKL) in the tumor/bone microenvironment. The CSR consists of a receptor activator of nuclear factor κ B (RANK, TNFRSF11A) ectodomain fused to a CD27 endodomain. Coexpression of this CSR in γδ CAR-T cells resulted in enhanced AP-1 transcriptional activation and improved γδ T-cell survival in vivo.

Together, these findings highlight the fundamental biological differences between γδ and αβ T cells and underscore the need for subset-specific engineering strategies. Tailoring chimeric receptor constructs to the unique signaling and metabolic profiles of γδ T cells is essential for fully harnessing their therapeutic potential, especially in the context of solid tumor immunotherapy.

## Results

### γδ CAR-T cells produce lower cytokine levels than αβ CAR-T cells do

**Fig. 1:**
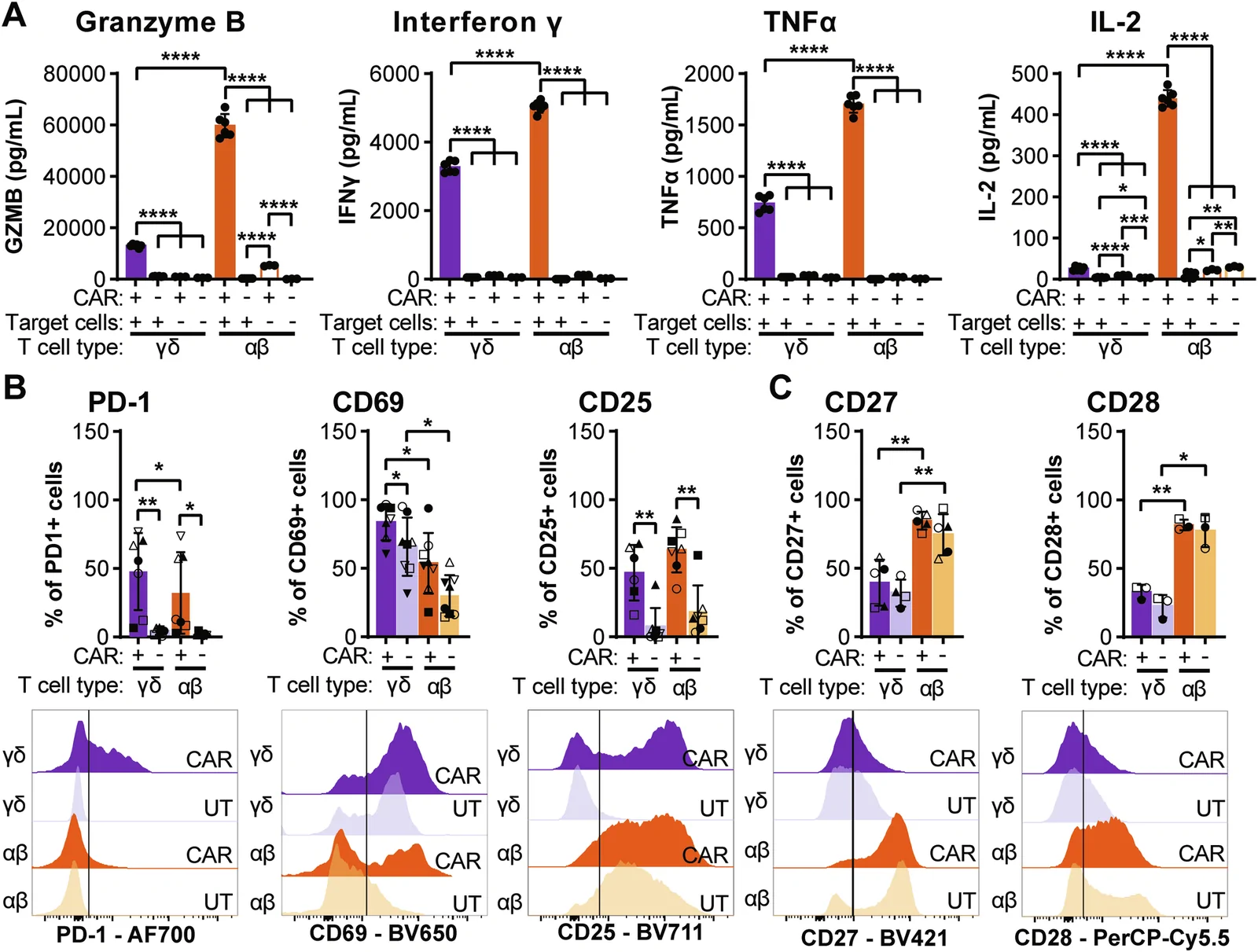
γδ and αβ CAR-T cells differ in phenotype and function. **A** Cytokine secretion by γδ and αβ T cells following overnight coculture with PSCA-expressing targets (C4-2B) at a 1:1 ratio. Quantification of cytokines in coculture supernatants was performed using the ELLA platform. **B** Surface expression (mean ± SD) of the activation markers PD-1, CD25, and CD69 and **C** the costimulatory markers CD28 and CD27 in resting untransduced (UT) and CAR-T cells; each symbol represents T cells from an independent healthy donor. Representative flow cytometry histograms are shown below. ^*^p < 0.05, ^**^p < 0.01, ^***^p < 0.001, ^****^p < 0.0001 (**A**) Unpaired *t* test, (**B**, **C**) paired *t* test. (**B**, **C**) were gated on lymphocytes/singlets/live/CD3+/either Vδ2+ or αβTCR+ cells

To define the functional properties of γδ CAR-T cells, we compared their effector cytokine secretion with that of αβ CAR-T cells derived from the same donor. We used a prostate stem cell antigen (PSCA)-targeted second-generation CAR design [21] as our main experimental model. This CAR contains a CD8α-derived hinge/transmembrane domain fused to a CD28 costimulatory moiety and a CD3ζ activation domain (herein named 8t28z). In our previous work, we showed that γδ and αβ CAR-T cells display comparable cytolytic strength against prostate cancer cell lines [21]. Expanding on these results, we found that upon overnight co-culture with PSCA-expressing C4-2B cells, CAR-induced secretion of granzyme B (GZMB), interferon-γ (IFNγ), tumor necrosis factor-α (TNFα), and interleukin-2 (IL-2) was observed for both T-cell subsets. However, cytokine levels were consistently and significantly lower in γδ CAR-T cells (Fig. 1A), even if their potential for polyfunctionality was comparable (Fig. S1A). Further phenotypical analysis revealed that compared with αβ CAR-T cells, γδ CAR-T cells expressed significantly higher levels of programmed cell death protein 1 (PD-1; *p* = 0.0477) and CD69 (*p* = 0.0147). In contrast, the expression of the activation marker CD25 was similar between subsets but appeared to be greater in CAR-expressing cells of both kinds (Fig. 1B). Importantly, compared with αβ CAR-T cells, γδ CAR-T cells expressed lower levels of the costimulatory molecules CD27 (*p* = 0.0143) and CD28 (*p* = 0.0039) (Fig. 1C). Differences between γδ and αβ CAR-T cells were not explained by differences in CAR expression, which varied among donors (range: 20–72%) but were comparable between T-cell subsets (Fig. S1B). These results indicate that while they are comparable in terms of their cytolytic potential, γδ and αβ CAR-T cells differ in terms of other important functional traits, warranting a deeper mechanistic analysis of their immunobiology.

### CAR activation induces differential signal transduction in γδ and αβ CAR-T cells

**Fig. 2:**
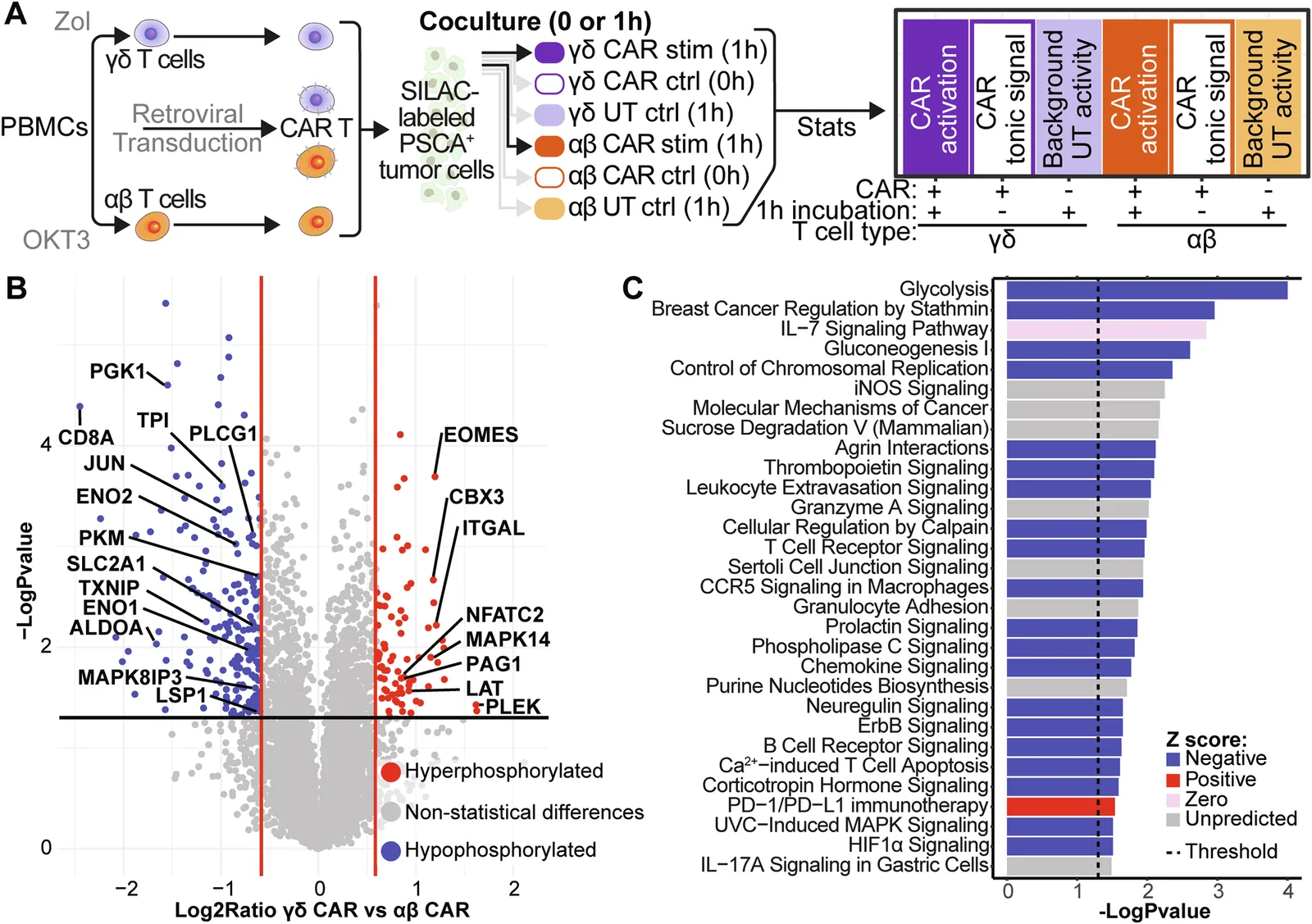
CAR-driven phosphorylation signals differ between γδ and αβ CAR-T cells. **A** Experimental design for testing differences in CAR-induced phosphorylation between γδ and αβ CAR-T cells. Zol: Zoledronic acid, ctrl: Control, stim: Stimulated. **B** Volcano plot showing phosphosite differential abundances in activated (1 h) γδ CAR-T cells compared with activated (1 h) αβ CAR-T cells (Welch’s *t* test *p* values < 0.05, |FC| > 1.5). **C** The top 30 significantly different canonical pathways that are overrepresented among the differentially phosphorylated proteins. *Z* scores were calculated by comparing the phosphorylation sites of γδ cells and αβ CAR-T cells

Prompted by the phenotypic differences observed between γδ and αβ CAR-T cells, we designed an approach to capture a snapshot of the signaling events induced by CAR activation in either cell subset. C4-2B PSCA^+^ cells were cultured in stable isotope labeling by amino acids in cell culture (SILAC) media until the incorporation of labeled amino acids reached more than 95% of the total proteome. These labeled tumor cells were co-cultured with unlabeled CAR- or untransduced (UT) T cells as background controls under three different conditions: (1) CAR-T cells incubated with tumor cells for 1 h (antigen-specific CAR-driven signaling); (2) CAR-T cells mixed with tumor cells without incubation (antigen-independent CAR-driven signaling or tonic signaling); and (3) UT-T cells incubated with tumor cells for 1 h (basal phosphorylation levels; Figs. 2A and S2).

Phosphopeptide abundance was quantified via tandem mass spectrometry, and statistical comparisons among groups were performed on the basis of a fold change >1.5 and a *p* value < 0.05 (Welch’s *t* test). A comparison of activated CAR-T cells (condition 1) revealed 307 phosphosites whose abundance significantly differed; 224 of them were hypophosphorylated in γδ CAR-T cells (Fig. 2B). Both CD8a and CD4 were among the hypophosphorylated events in γδ CAR-T cells, which is consistent with lower (CD8a) or null (CD4) coreceptor expression in γδ T cells [36, 37] (Fig. 2B). Hypophosphorylated proteins included phospholipase C Gamma 1 (PLCγ1, PLCG1), Jun proto-oncogene (c-Jun, JUN), solute carrier family 2 facilitated glucose transporter member 1 (GLUT-1, SLC2A1), among others. Hyperphosphorylated proteins included nuclear factor of activated T cells, cytoplasmic 2 (NFAT1, NFATC2), mitogen-activated protein kinase 14 (MK14, MAPK14), phosphoprotein associated with glycosphingolipid-enriched microdomains 1 (PHAG1, PAG1), integrin subunit alpha L (LFA-1, ITGAL), and eomesodermin homolog (EOMES), among others. A full list of differentially phosphorylated proteins can be found in Table S1.

Pathway enrichment analysis revealed 55 potential canonical pathways that were significantly overrepresented among the differentially phosphorylated events. The 30 most significant canonical pathways are represented in Fig. 2C. Notably, pathways related to glycolysis/gluconeogenesis and T-cell receptor (TCR) signaling were among the most enriched, with a negative Z score, suggesting that these pathways are more strongly activated in αβ than in γδ CAR-T cells. Since CAR-T-cell activation in this assay was driven solely through the CAR and not the endogenous TCR, these results suggest that the same CAR construct can drive different activation signals depending on the T-cell type in which it is expressed.

### γδ T cells display lower metabolic activity than αβ T cells do

**Fig. 3:**
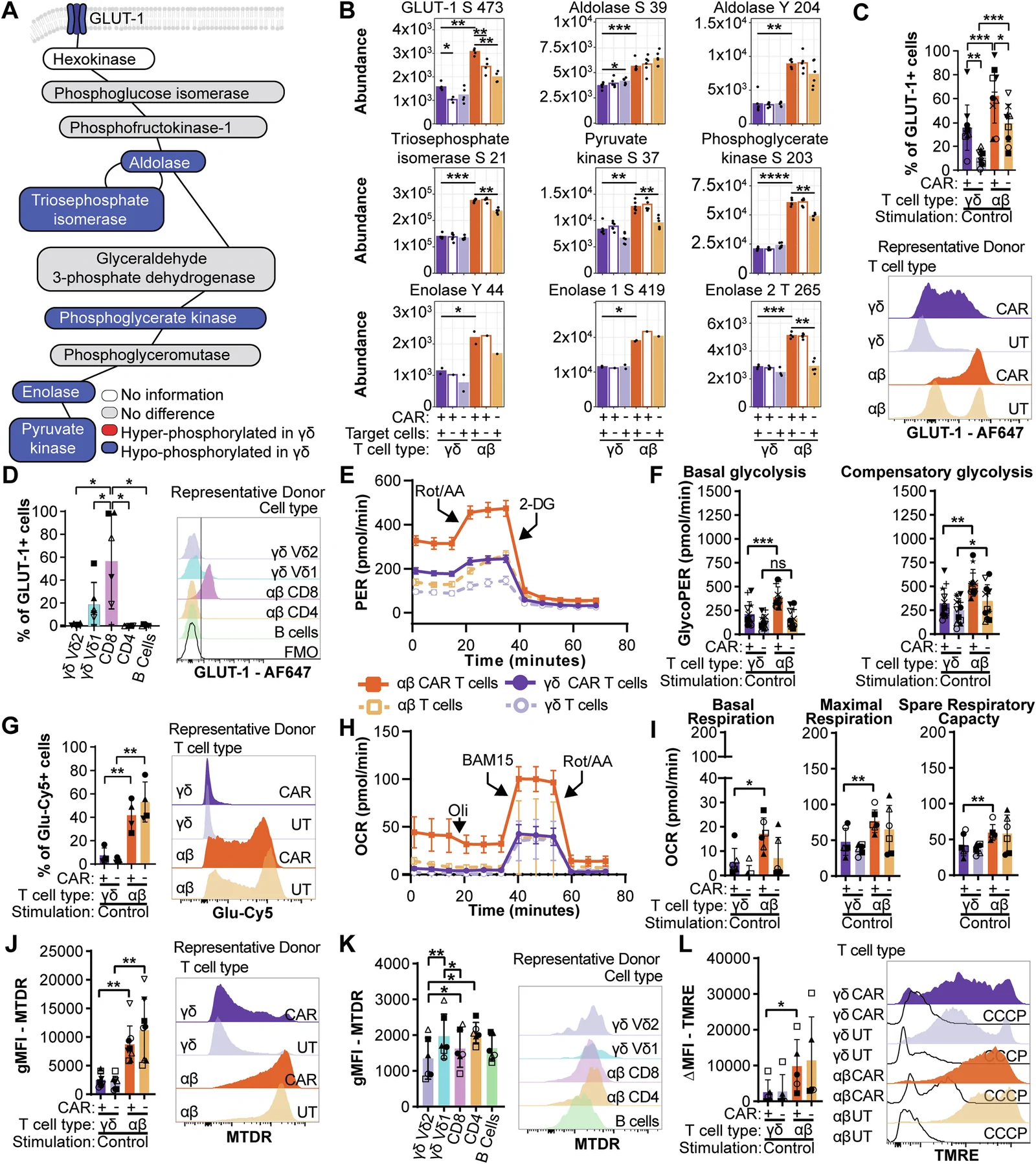
Compared with αβ cells, γδ CAR-T cells have lower metabolic activity. **A** Diagram of the glycolytic pathway highlighting enzymes that have differential phosphorylation. **B** Phosphosite abundance in activated γδ and αβ CAR-T cells (Welch’s *t* test, *p* < 0.05, |FC| > 1.5). **C** GLUT-1 surface expression in unstimulated T cells (*n* = 8) and **D** noncultured PBMCs (*n* = 5). **E** Proton efflux rate (PER) of unstimulated T cells. Representative donor. Rot/AA: rotenone and antimycin A; 2DG: 2-deoxy-D-glucose. **F** Basal and compensatory glycolysis of unstimulated T cells derived from the glycolytic proton efflux rate (GlycoPER) (*n* = 12). **G** Glucose-Cy5 (Glu-Cy5) uptake by unstimulated T cells (*n* = 4). **H** Oxygen consumption rate (OCR) of unstimulated T cells. Representative donor. Oli: Oligomycin, BAM15: C_16_H_10_F_2_N_6_O. **I** Basal, maximal and spare respiratory capacity (SRC) of unstimulated T cells obtained from the OCR (*n* = 6). **J** MitoTracker™ DeepRed (MTDR) geometric mean fluorescence intensity (gMFI) of unstimulated T cells (*n* = 8) and **K** noncultured PBMCs (*n* = 6). **L** MFI of tetramethylrhodamine (TMRE) as an indirect measurement of the mitochondrial membrane potential of unstimulated T cells (*n* = 5). Representative flow cytometry histograms are depicted alongside the relevant bar chart. The data in (**C**), (**G**), (**J**), and (**L**) reflect lymphocytes/singlets/live/CD3+/Vδ2+ or αβTCR+ gates, while those in (**D**) and (**K**) reflect lymphocytes/singlets/live/CD45+/either CD19+, CD3+/αβTCR+ , CD3+/Vδ1+, or CD3+/Vδ2+. Symbols from each bar (mean ± SD) in (**C**, **D**), (**F**, **G**), and (**I**–**L**) represent independent healthy donors. ^*^p < 0.05, ^**^p < 0.01, ^***^p < 0.001, and ^****^p < 0.0001 (**B** Welch’s *t* test; **C**, **D**, **F**, **G**, **I**–**L** paired *t* test)

Glycolysis and gluconeogenesis signaling networks were the first and fourth most overrepresented pathways, respectively,  among the differentially phosphorylated proteins (Fig. 2C). Five enzymes involved in these pathways were less phosphorylated in γδ CAR-T cells (Fig. 3A), along with the main glucose transporter GLUT-1 (SLC2A1, S473 *p* = 0.00613). The phosphorylation levels of aldolase (ALDOA, S39 *p* = 0.00062; Y204 *p* = 0.00929), triosephosphate isomerase (TPI1, S21 *p* = 0.00025), phosphoglycerate kinase (PGK1, S203 *p* = 0.00002), pyruvate kinase (PKM, S37 *p* = 0.03398), and enolase (ENO1, Y44 *p* = 0.03398; S419 p = 0.01140; ENO2, T265 *p* = 0.00094) differed significantly between γδ and αβ CAR-T cells (Fig. 3B). These differences appeared to be intrinsic to the cell subset because similar trends were observed when CAR-negative γδ cells were compared with αβ T cells or CAR-expressing cells in the absence of antigenic stimulation (Fig. 3B). We next quantified the messenger RNA (mRNA) abundance of hexokinase, phosphofructokinase, and pyruvate kinase in γδ and αβ T cells by reverse transcription polymerase chain reaction (RT‒PCR) to test whether the differences in phosphorylation were due to differences in gene expression. As shown in Fig. S3A, gene expression was comparable among cell types, suggesting that the observed differences in phosphorylation were due to differences in the activation status of the respective pathways. In contrast, GLUT-1 phosphorylation was lower at S473 (Fig. 3B), and its protein expression was lower (Fig. 3C). We analyzed GLUT-1 surface expression in the UT and CAR-T cells, either in the resting state or following activation (anti-CD3 clone: OKT3, 24 h; Fig. S3B). GLUT-1 was expressed less frequently in γδ than in αβ UT T cells (*p* = 0.0001). Moreover, CAR expression was associated with increased expression of GLUT-1 in both subsets (UT vs CAR in γδ, *p* = 0.0015; in αβ, *p* = 0.0174). Despite the CAR-associated increase in GLUT-1 expression, a significant difference in expression between γδ and αβ CAR-T cells remained (*p* = 0.0005; Fig. 3C). After OKT3 activation, GLUT-1 expression increased slightly under all the conditions tested; however, the differences between γδ and αβ CAR-T cells were still significant (*p* = 0.0107; Fig. S3B). To test whether the lower expression of GLUT-1 observed in γδ T cells was a result of ex vivo culture, we analyzed its expression in uncultured peripheral blood mononuclear cells (PBMCs; Fig. 3D). On average, 56.27% of the CD8 T cells expressed GLUT-1, which was significantly greater than the average expression in Vδ2 T cells (1.15%, *p* = 0.0208), Vδ1 T cells (18.87%, *p* = 0.0250), and even CD4 T cells (0.03%, *p* = 0.0209).

We also observed a trend (*p* = 0.0681) toward greater expression of GLUT-1 in Vδ1 T cells than in circulating Vδ2 T cells (Fig. 3D). To test whether the observed lower expression of GLUT-1 was compensated by the expression of other glucose transporters previously reported in peripheral blood lymphocytes, we analyzed the expression of GLUT-3, GLUT-4, GLUT-6, and GLUT-8 by flow cytometry [38–40] (Fig. S3C). No statistically significant difference in the expression of either transporter among T-cell subsets was observed, suggesting that γδ T cells may be less capable of glucose uptake than their αβ counterparts.

Next, we sought to elucidate the functional implications of the observed differences in the phosphorylation of glycolytic enzymes and GLUT-1 expression. We first measured the glycolytic rates of the different T-cell subsets using the SeaHorse XF Glycolytic Rate Assay Kit (Figs. 3E and S3D). In agreement with previous reports, we observed great variability in the glycolytic rate among donors [41]. Despite this variability, we found that while the basal glycolytic rates of resting γδ and αβ UT T cells were comparable, the basal glycolytic rate of γδ CAR-T cells was significantly lower than that of αβ CAR-T cells (*p* = 0.002; Fig. 3F). Compensatory glycolysis, which is considered the maximal glycolytic capacity that cells can achieve under extreme conditions, was significantly lower in both γδ-UT (*p* = 0.0359) and γδ-CAR (*p* = 0.0037) T cells than in their corresponding αβ counterparts (Fig. 3F). When T cells were stimulated with OKT3, both basal and compensatory glycolytic rates were greater in αβ T cells (Fig. S3E). Consistently, the results of glucose uptake experiments revealed that compared with αβ T cells, γδ T cells take up less glucose independent of CAR expression (γδ vs αβ UT *p* = 0.0071; γδ vs αβ CAR *p* = 0.0049; Fig. 3G).

In addition to producing energy for homeostatic processes, glycolysis is particularly relevant for the acute effector functions of T lymphocytes [42, 43]. In contrast, oxidative phosphorylation is more dominant in resting lymphocytes, prominently in naïve and central memory cells [44, 45]. To test whether the lower glycolytic rate observed in γδ T cells was compensated by greater oxidative capacity, we quantified oxygen consumption using a Seahorse XF T-Cell Metabolic Profiling Kit (Figs. 3H and S3F). We found that basal respiration, measured by the oxygen consumption rate (OCR) before the electron chain was altered, was significantly lower in unstimulated γδ CAR-T cells than in αβ CAR-T cells (*p* = 0.0326; Fig. 3I). This pattern was also observed for maximal respiration (p = 0.0081; Fig. 3I) and for spare respiratory capacity (SRC) (*p* = 0.0075; Fig. 3I). Following activation, the differences in basal respiration and the SRC between T-cell subsets were not statistically significant (Fig. S3G). However, compared with their αβ counterparts, both CAR (*p* = 0.0399) and UT (*p* = 0.0497) γδ T cells exhibited lower maximal oxygen consumption (Fig. S3G).

We next analyzed the mitochondrial mass using MitoTracker Deep Red (MTDR) dye. In line with previous findings, compared with αβ T cells, γδ T cells contained less mitochondrial mass (γδ vs αβ UT *p* = 0.0060; γδ vs αβ CAR *p* = 0.0046; Fig. 3J), and neither CAR expression nor OKT3 activation affected the mitochondrial mass content of either cell subset (Fig. S3H). Moreover, compared with CD4 (*p* = 0.0151), CD8 (p = 0.0389), and Vδ1 (*p* = 0.001) T cells, uncultured Vδ2 T cells from PBMCs had lower mitochondrial mass (Fig. 3K), indicating that these properties are cell intrinsic and not a product of ex vivo manipulation. Finally, the membrane potential of γδ CAR-T cells was lower than that of αβ CAR-T cells, as evaluated by tetramethylrhodamine, ethyl ester (TMRE) staining (*p* = 0.0214; Figs. 3L and S3I). Taken together, these results indicate that the two major mechanisms of ATP production (namely, glycolysis and oxidative phosphorylation) are less active in γδ than in αβ CAR-T cells. These differences were potentially due to decreased mitochondrial mass.

### TXNIP inhibition improves γδ T-cell metabolism and function

**Fig. 4:**
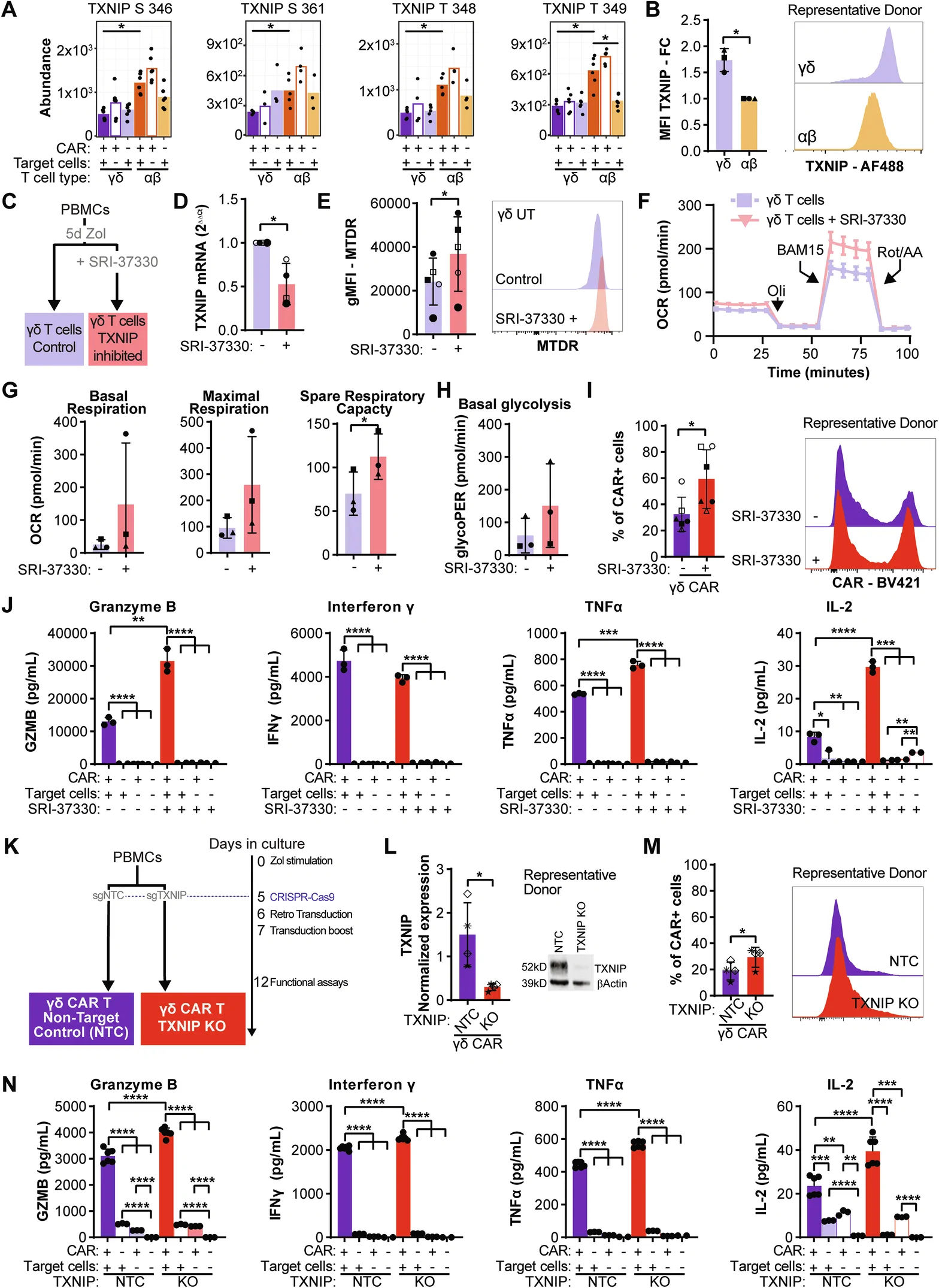
TXNIP inhibition enhances γδ T-cell products. **A** Phosphorylation abundance of TXNIP phosphosites in γδ and αβ CAR-T cells. **B** Intracellular TXNIP expression in unstimulated T cells (*n* = 3). **C** Alternative γδ T-cell expansion protocol incorporating SRI-37330 (5 μM) with medium changes every other day. **D** Relative TXNIP abundance (RT‒qPCR) in γδ T cells expanded with the new protocol (*n* = 3). **E** Comparison of T cell mitochondrial mass based on gMFI of MTDR staining, between expansion methods (*n* = 5). **F** Representative OCRs of expanded γδ T cells. **G** Basal, maximal, and spare respiratory capacity (SRC) derived from the OCR measurements (*n* = 3). **H** GlycoPER as an indirect indicator of basal glycolysis (*n* = 3). **I** Surface CAR expression in γδ T cells expanded under control or TXNIP-inhibited conditions (*n* = 6). **J** Effector molecule secretion by γδ UT and CAR-T cells after overnight coculture with C4-2B PSCA^+^ cells. **K** γδ T-cell expansion protocol adapted to incorporate CRISPR-Cas9 of TXNIP or a nontargeting single-guide RNA (NTC) as a control. **L** Normalized TXNIP expression 7 days post-knockout (*n* = 4). Representative WB results are shown on the right. **M** Surface CAR expression in control (NTC) or TXNIP-KO γδ T cells (*n* = 4). **N** Effector molecule secretion by TXNIP KO and control γδ UT and CAR-T cells after overnight coculture with C4-2B PSCA^+^ cells. ^*^p < 0.05, ^**^p < 0.01, ^***^p < 0.001, ^****^p < 0.0001 (**A** Welch’s *t* test; **B**, **D**, **E**, **G**, **I**, **L**, **M** paired *t* test;** J**, **N** unpaired *t* test). The symbols from each bar in **B**, **D** (fold change ± SD), **E**, **G**–**I**, **L**, and **M** (mean ± SD) represent independent healthy donors. **B** Data reflect singlets/live/CD3+/either Vδ2+ or αβTCR+ obtained by ImageStream; **E**, **I**, **M** Data reflect lymphocytes/singlets/live/CD3+/Vδ2+ obtained by flow cytometry; representative histograms are shown. Analysis in **D**–**H** was performed on day 5 after expansion

Among the proteins whose phosphorylation differed between γδ and αβ CAR-T cells (Fig. 2B), thioredoxin-interacting protein (TXNIP) emerged as a potential actionable target for enhancing T-cell metabolism. TXNIP is a multifunctional protein involved in the cellular oxidative stress response and glucose metabolism [46]. Notably, all the identified TXNIP phospho-sites were less abundant in γδ CAR-T cells (S346 *p* = 0.01522, S361 *p* = 0.03584, T348 p = 0.00557, T349 *p* = 0.01817; Fig. 4A). In particular, the phosphorylation of T349 triggers the proteasomal degradation of TXNIP [47]. Thus, the decreased phosphorylation of TXNIP in γδ T cells suggested that these cells may accumulate TXNIP because of reduced degradation. To validate this prediction, we analyzed TXNIP expression in cultured T cells and reported that compared with αβ T cells, γδ T cells express ~1.74 (±0.22) times more TXNIP (*p* = 0.0068; Fig. 4B). We hypothesized that TXNIP accumulation may inhibit glycolysis and lead to mitochondrial damage, which could be mitigated through pharmacological inhibition of TXNIP [48, 49]. To test this concept, we designed a new γδ T-cell expansion protocol that incorporated SRI-37330, a TXNIP inhibitor [50], during ex vivo culture (Fig. 4C). After 5 days of γδ T-cell expansion, we analyzed the expression of TXNIP mRNA by RT‒PCR and observed a reduction in TXNIP transcription in the cells treated with SRI-37330 (Fig. 4D). TXNIP inhibition was associated with increased mitochondrial mass (*p* = 0.0243; Fig. 4E), a significant increase in spare respiratory capacity (*p* = 0.0172; Fig. 4F, G), and increased basal glycolytic rates (Fig. 4H). To test the effect of TXNIP on CAR-T-cell function, we generated γδ CAR-T cells in the presence of the TXNIP inhibitor during manufacturing. Compared with the control cells (expanded without the inhibitor), γδ T cells expanded with SRI-37330 showed a greater percentage of CAR expression r (*p* = 0.0085; Fig. 4I). We then evaluated the activation and effector function of these CAR-T cells by ELLA (next-generation automated immunoassay platform) following coculture with PSCA-expressing C4-2B cells. γδ CAR-T cells expanded with SRI-37330 released significantly more TNFα, GZMB, and IL-2 (Fig. 4J), indicating an improvement in effector function associated with TXNIP inhibition. We further validated these findings using CRISPR-Cas9-mediated TXNIP knockout during γδ CAR-T-cell generation (Fig. 4K). Compared with nontargeting control (NTC) cells, TXNIP-KO cells showed reduced TXNIP expression 7 days post-electroporation (*p* = 0.0373; Fig. 4L). Consistent with our pharmacological inhibition results, the percentage of CAR-expressing cells was greater in TXNIP-KO T cells than in NTC control T cells (*p* = 0.0218; Fig. 4M). Following coculture with PSCA-expressing C4-2B cells, ELLA analysis revealed significantly greater release of TNFα, GZMB, IFNγ, and IL-2 by TXNIP-KO γδ CAR-T cells than by NTC control cells (Fig. 4N). Collectively, these results suggest that TXNIP plays important roles in modulating metabolism and effector function in γδ CAR-T cells and may represent an actionable target for enhancing cellular immunotherapies.

### CAR activation triggers different TCR signaling events in γδ and αβ CAR-T cells

**Fig. 5:**
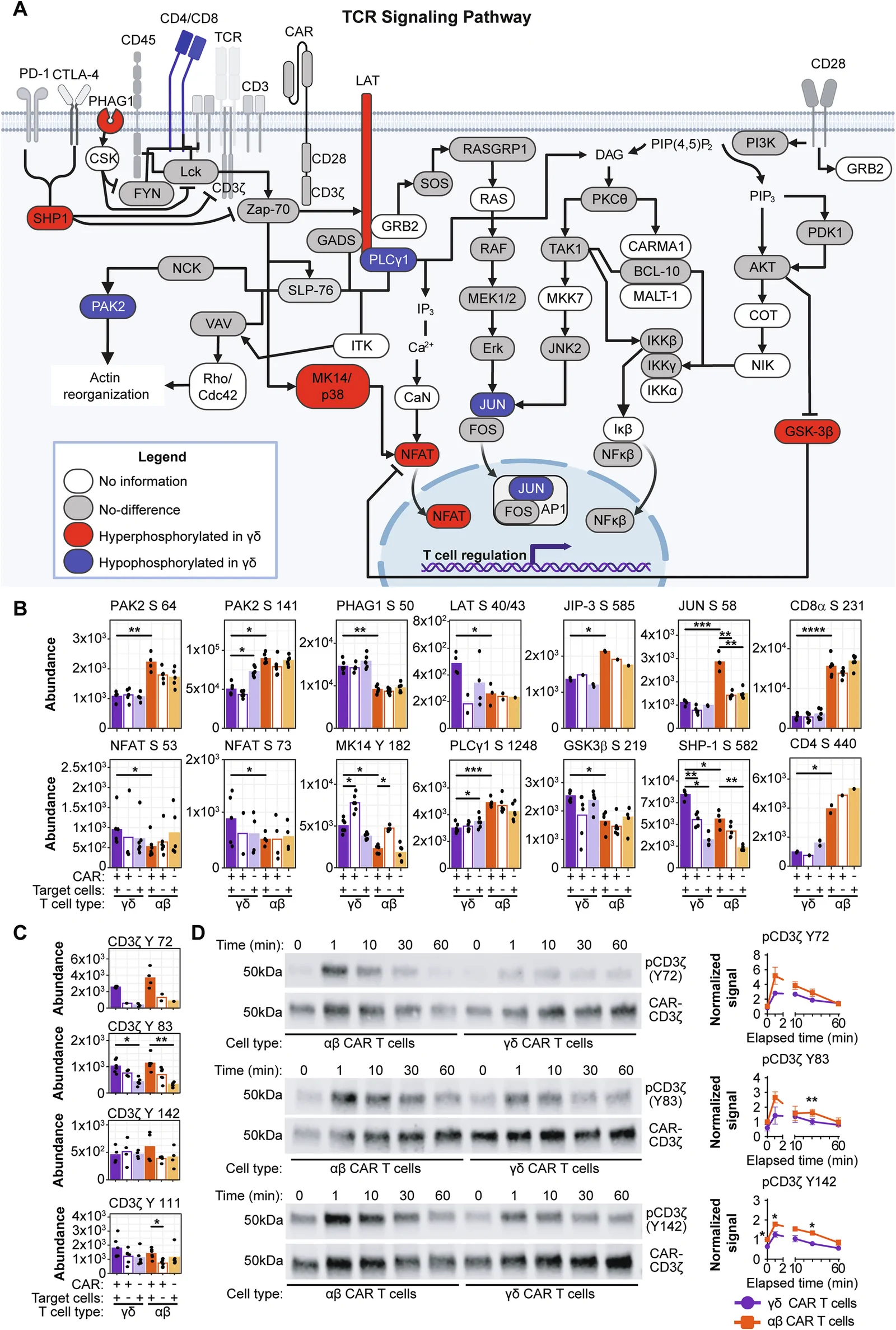
CAR-driven Signaling downstream of the TCR differs between γδ and αβ CAR-T cells. **A** Diagram of the canonical molecules involved in TCR signaling, outlining differences in phosphorylation between activated γδ and αβ CAR-T cells. **B** Phosphorylation abundance of the individual phosphosites corresponding to the proteins highlighted in **A**. **C** Abundance of phosphorylated tyrosine sites within the CD3ζ ITAMs in resting and activated CAR-T cells. **D** Time-course analysis of CAR phosphorylation at tyrosine sites corresponding to CD3ζ ITAMs (top, pY72; middle, pY83; bottom, pY142). CAR was immunoprecipitated following stimulation with C4-2B PSCA^+^ cells (0 to 60 min); the band signal was normalized at each time point to that of total CAR-CD3ζ. The average signal from three independent donor replicates, normalized to the αβ CAR-T-cell signal at time zero, is presented on the right. **B**, **C** ^*^p < 0.05, ^**^p < 0.01, ^***^p < 0.001, ^****^p < 0.0001 (Welch’s *t* test); **D** ^*^p < 0.05, ^**^p < 0.01 (paired *t* test)

In addition to glycolysis and gluconeogenesis, TCR signaling was among the top ten most overrepresented canonical pathways identified in our phosphoproteomic experiment (Fig. 2C). In the context of our experiment, we attributed the activation of this pathway to the signal provided by the CAR molecules (designed to mimic TCR engagement). Upon stimulation of CAR-T cells with live tumor cells for 1 h, the phosphorylation levels of early signaling kinases such as tyrosine-protein kinase ITK/TSK (ITK), leukocyte c-terminal src kinase (LCK), and 70 kDa zeta-chain associated protein (ZAP70) were comparable across both cell types (Fig. 5A), suggesting that proximal activation thresholds were achieved in both cell subsets. However, differences in activation were evident downstream, as the phosphorylation of the transcription factor NFAT (NFATC2, S53 *p* = 0.01981; S73 *p* = 0.0319) was significantly greater and the phosphorylation of AP-1 (JUN, S58 p = 0.00062) was significantly lower in γδ CAR-T cells (Fig. 5A). p21-Activated kinase 2 (PAK2), a kinase involved in cell adhesion and associated with IL-2 production [51], was hypophosphorylated at activation site S141 (*p* = 0.01581) in γδ CAR-T cells (Fig. 5B), suggesting diminished PAK2 activity in this subset. In contrast, decreased phosphorylation of PLCγ1 (PLCG1) at its inhibitory site, S1248 [52] (*p* = 0.00082), suggested increased enzymatic activity in γδ CAR-T cells (Fig. 5B). PLCγ1 is a critical effector of diacylglycerol (DAG)- and inositol trisphosphate (IP₃)-mediated calcium signaling, and its activity can modulate the activation of the transcription factor NFAT. MK14 (also known as p38) was differentially phosphorylated at its activation site, Y182 (*p* = 0.01257; Fig. 5B), which can partially account for the hyperphosphorylated state of NFAT found in γδ CAR-T cells (Fig. 5A) [53, 54].

The low detection of CD4 (*p* = 0.01389) and CD8α (CD8A, *p* = 0.00004) phosphorylation in γδ CAR-T cells is compatible with the lower or null expression of coreceptors in this cell subset (Fig. 5B). Additional differences in phosphorylation, i.e., PAK2 S64 (p = 0.00797), c-Jun-amino-terminal kinase-interacting protein 3 (JIP3) S585 (MAPK8IP3, p = 0.02531), JUN S58, PHAG1 S50 (PAG1, *p* = 0.00933), linker for activation of T-cell family member 1 (LAT) S40/43 (*p* = 0.02706), NFAT1 S53/S73 (NFATC2, *p* = 0.01551), glycogen synthase kinase-3 beta (GSK3β, *p* = 0.016) S219, and SHP-1 S582 (PTPN6, *p* = 0.03806), were also noted (Fig. 5B), yet the functional implications of these sites remain unclear because of limited annotation in the literature.

Notably, phosphorylation at CD3ζ Y83 (CD247) was significantly greater in CAR-T cells than in their UT counterparts (Fig. 5C, γδ CAR vs UT *p* = 0.01229, and αβ CAR vs UT *p* = 0.00539). These results indicate that the CAR activation domain is phosphorylated following stimulation. However, we cannot rule out that this activation is due to tonic signaling because such elevation was not significant between stimulated and unstimulated CAR-T cells when analyzed 1 h post-stimulation. A similar pattern was observed for the phosphorylation of CD3ζ Y72 (Fig. 5C). In contrast, the CD3ζ Y111 residue was significantly more phosphorylated in stimulated CAR-T cells than in nonstimulated CAR-T cells, but only in the αβ subset (Fig. 5C). Interestingly, the levels of these phospho-sites were comparable between the subsets when activated CAR-T cells were compared at 60 min after antigen engagement.

To gain a deeper understanding of the kinetics of CAR activation, we expanded the phosphoproteomics results by conducting a targeted time-course analysis of CD3ζ phosphorylation between 1 and 60 min post-stimulation. We examined the phosphorylation of the immunoreceptor tyrosine-based activation motif (ITAM) within CD3ζ by Western blot (WB) across three independent donors. Phosphorylation of Y72, Y83, and Y142 peaked at 1 minute post-stimulation in both T cell subsets. Consistent with previous observations, ITAM phosphorylation was comparable between γδ and αβ CAR-T cells at the 1-h time point (Fig. 5D). However, differential phosphorylation was detected for Y83 (*p* = 0.0046 at 30 min) and Y142 (p = 0.0197 at 0 min, *p* = 0.03 at 10 min and *p* = 0.0119 at 30 min), where the phosphorylation of the γδ CAR-T cells was attenuated relative to that of their αβ counterparts over time (Fig. 5D). Taken together, these findings suggest that while early activation thresholds are met across both CAR-T-cell types, γδ T cells integrate antigen signals with different kinetics or magnitudes, resulting in differential downstream signaling in pathways of proven functional relevance.

### CD27 costimulation delivered through a chimeric switch receptor improves AP-1 activation and T-cell survival

**Fig. 6:**
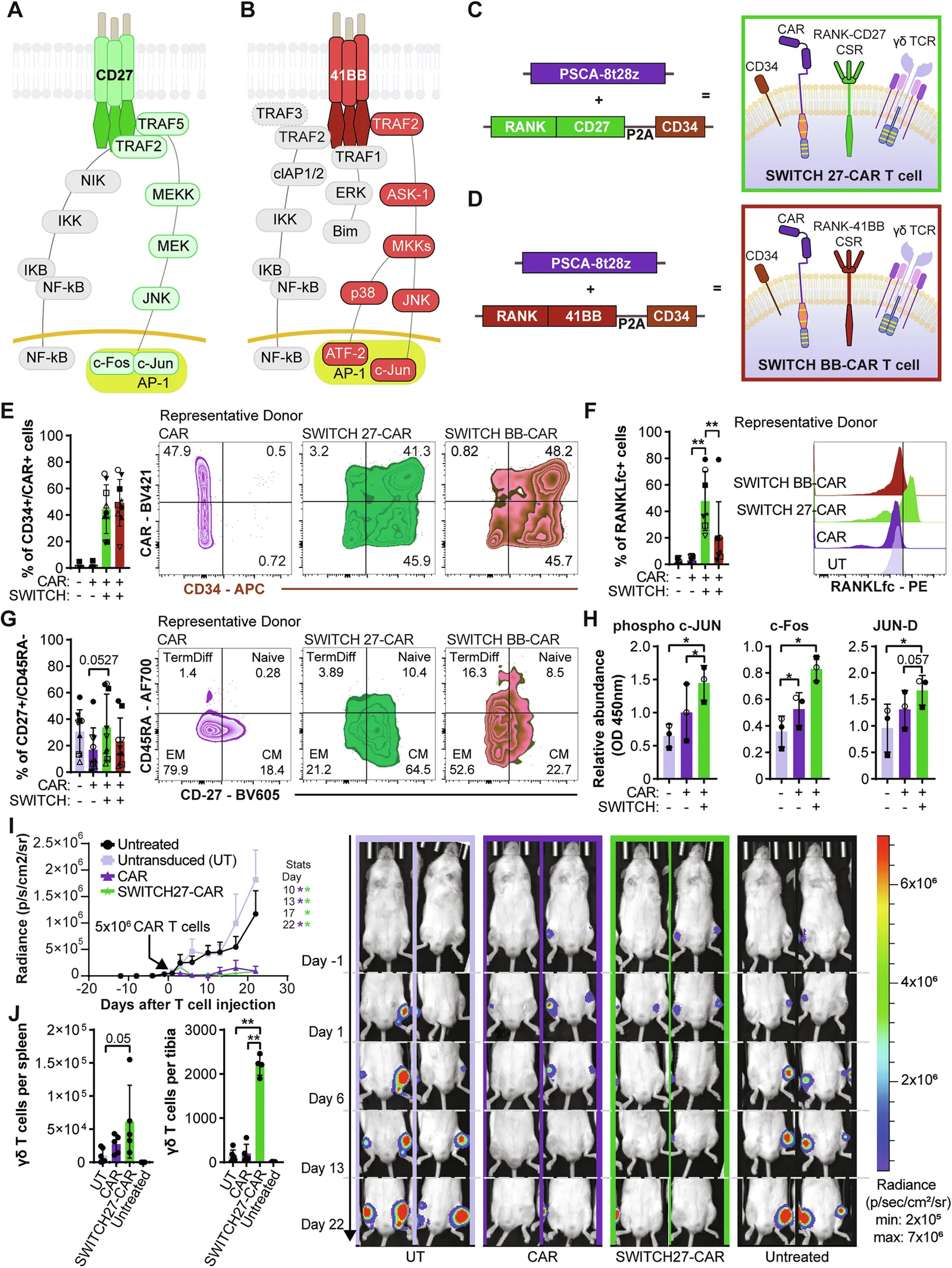
SWITCH27 rescues AP-1 signaling and increases γδ CAR-T-cell persistence. **A** Diagram of the CD27 and **B** 41BB signaling pathways. Diagram of the (**C**) RANK-CD27 and (**D**) RANK-41BB chimeric switch receptors used to cotransduce γδ T cells into SWITCH27-CAR and SWITCHBB-CAR-T cells. **E** Proportion of CAR+/CD34+ γδ T cells (*n* = 9 independent donors). **F** Surface binding of RANKL-Fc to γδ T cells (*n* = 7 independent donors). **G** Frequency of CD27⁺CD45RA⁻ γδ T cells (central memory phenotype, *n* = 9 independent donors). **H** Relative abundance of AP-1 proteins in the nuclei of γδ T cells (*n* = 3 independent donors). **I** The mean bioluminescence of tumors (photons×seconds^−1^×centimeter^−2^×steradian^−1^) from untreated (PBS injection), γδ untransduced (UT), γδ CAR, or γδ SWITCH27-CAR-Treated tumor-bearing mice (*n* > 5 mice/group). Representative images are shown. γδ CAR-T cells were delivered on day 0 (arrow). Significant differences per day are shown on the right; a complete matrix of *p* values is included in Fig. S6C. **J** Total γδ T cells recovered from the spleen and tibial bone marrow of treated mice. Symbols from each bar (mean ± SD) in (**E**–**H**) represent independent healthy donors. The data in (**E**–**G**) and (**J**) represent lymphoid/singlet/live/CD3 + /Vδ2+ cells, and representative flow cytometry histograms are shown. ^*^p < 0.05, ^**^p < 0.01 (**F**‒**H**, paired *t* test; **J** unpaired *t* test; **I** Welsh *t* test of the CAR (purple) or SWITCH27-CAR (green) against the untreated control)

On the basis of the observation of reduced JUN phosphorylation in γδ CAR-T cells (Fig. 5A), we postulated that supplementation with JUN signaling may harness their functionality and/or persistence. Unlike similar approaches involving JUN overexpression [55, 56], we sought to increase JUN signaling through the delivery of complementary costimulatory signals. More specifically, we proposed that CD27- or 41BB-triggered signaling cascades converge on the activation of AP-1 (JUN/FOS) and nuclear factor kappa-B (NFĸB; Fig. 6A, B). CD27 is of particular interest because we observed a significant expression deficit in γδ compared with that in αβ CAR-T cells (Fig. 1C). To achieve CSR activation within the tumor, we leveraged the overexpression of RANKL (TNFSF11) in the bone/tumor microenvironment [57]. We fused CD27 antigen (CD27) or tumor necrosis factor receptor superfamily member 9 (41BB, TNFRSF9) signaling domains with the ectodomain of the receptor of RANKL, RANK (TNFRSF11A), with the goal of converting a pro-osteolytic input signal (RANKL) into a costimulatory output signal. We cloned each CSR in a retroviral vector, together with a truncated version of hematopoietic progenitor cell antigen CD34 (tCD34), to be used as a marker of transduction. Through the cotransduction of our CAR vector with either the CD27- or 41BB-containing CSR, we aimed to generate the corresponding γδ SWITCH27 or SWITCHBB-CAR-T cells (Fig. 6C, D). Cotransduction of the PSCA-8t28z CAR with either CSR vector yielded ~50% double-positive cells on average (*n* = 9 independent donors; Fig. 6E). We next tested the ability of SWITCH-CAR-T cells to bind to a soluble recombinant version of RANKL (RANKL-Fc). Although both versions of the CSR bound RANKL, the percentage of T cells that bound RANKL was significantly greater for the SWITCH27 construct (*p* = 0.0051; Fig. 6F). Interestingly, we detected an increased frequency of CD27^+^/CD45RA^-^ cells in the SWITCH27-CAR-T cells (*p* = 0.057; Fig. 6G) but not in the SWITCHBB-CAR-T cells. This central memory phenotype has been linked to increased expansion of γδ T cells from cancer patients [14, 58, 59]. On the basis of these results, we chose SWITCH27 for further analysis.

We next tested whether SWITCH27 engagement resulted in the activation of AP-1 signaling. To that end, we used a TransAM® AP-1 enzyme-linked immunosorbent assay (ELISA) kit to quantify the relative abundance of nuclear c-Fos, JUN-D, and phosphorylated c-JUN in activated T cells. Compared with UT T cells, γδ SWITCH27-CAR-T cells had a significantly greater abundance of all three AP-1 components (phospho c-JUN *p* = 0.0153, c-Fos *p* = 0.0492, JUN-D *p* = 0.0201), whereas γδ CAR-T cells showed increased c-Fos expression only (*p* = 0.0047; Fig. 6H). Importantly, γδ SWITCH27-CAR-T cells had a significantly greater abundance of phospho c-JUN than γδ CAR-T cells (*p* = 0.0497). Interestingly, αβ SWITCH27-CAR-T cells did not benefit from CSR expression in terms of AP-1 activation, although we confirmed CSR expression by flow cytometry (Fig. S6A, B). These results confirm that the CD27 costimulatory signaling provided by the new CSR increases JUN activity in γδ CAR-T cells in the presence of RANKL.

Finally, we analyzed the in vivo performance of SWITCH27-CAR-T cells using a murine model of bone-metastatic prostate cancer. Mice bearing intratibial xenografts of PSCA^+^ C4-2B cells were treated with systemic infusions of γδ CAR or γδ SWITCH27-CAR-T cells. Mice that received untransduced (UT) T cells or phosphate-buffered saline (PBS, Untreated) were used as negative controls (Fig. 6I). Both γδ CAR-T cellsand SWITCH27-CAR-T cells controlled tumor burden, demonstrating that a secondary receptor does not hinder CAR activity (Figs. 6I and S6C). Notably, endpoint analysis revealed a significantly greater abundance of γδ T cells in the bone marrow of γδ SWITCH27-CAR-treated mice compared to the CAR-T (*p* < 0.0001) or UT (*p* < 0.0001) groups Sγ (Fig. 6J), suggesting that enhanced in vivo persistence was mediated by CD27 costimulation. In the spleen, we observed a trend for greater cell numbers in the SWITCH-CAR group compared to UT (*p* = 0.05), but not compared to the CAR group, consistent with dependency on RANKL. A second experimental model consisting of a xenograft of U266 myeloma cells in NSG mice and then treatment with a single infusion of anti-B-cell maturation antigen (BCMA) γδ CAR-T cells partially recapitulated these results. As shown in Fig. S6D, E, we observed that γδ T cells expressing a second-generation CAR (BCMA-8t28z) alone or in combination with the SWITCH27 receptor achieved similar tumor control, with a trend toward superior persistence of SWITCH27-CAR-T cells in femur bone marrow (Fig. S6F). Additional studies need to be performed to quantify the benefit of the CSR for other cancer indications.

Collectively, these results demonstrate that the integration of a costimulatory signal through a separate CSR can improve γδ CAR-T-cell persistence without impairing antitumor function.

## Discussion

γδ T cells offer an attractive platform for adoptive cell therapy against solid tumors [1, 17, 60]. However, the translational potential of γδ T cells remains unclear because of a gap in knowledge concerning their unique immunobiology, including their signal transduction needs [1]. In this study, we conducted a systematic comparative analysis of CAR-triggered signaling pathways in γδ and αβ T cells and elucidated functional and mechanistic differences that can inform the design of next-generation γδ CAR-T-cell therapies.

Among the most notable differences across subsets, we found that compared with their αβ counterparts, γδ CAR-T cells produce significantly lower levels of proinflammatory cytokines such as IFNγ, GZMB, TNFα, and IL-2 despite showing comparable cytolytic potency [21]. This profile suggests that γδ CAR-T cells can mediate effective tumor control with a decreased risk of cytokine-associated toxicity. Activation markers such as PD-1 and CD69 are expressed at significantly higher levels in cultured γδ cells than in αβ CAR-T cells. These findings align with those of previous studies reporting that elevated PD-1 expression in γδ T cells is correlated with decreased cytokine release, particularly that of IFN-γ, following interaction with PD-L1-expressing targets [61–63]. Importantly, this functional attenuation does not compromise the cytotoxicity against zoledronate (Zol)-pretreated tumor cells, regardless of PD-L1 expression levels [61]. In our model, UT T cells did not express PD-1, which is consistent with the well-documented transient nature of PD-1 expression, which typically peaks early after activation and declines over time [27]. The CD69 expression dynamics observed in our study also mirrored previously reported patterns; Hoeres et al. reported that Zol and IL-2 treatment induced CD69 expression in ~80% of γδ T cells on day 2, which decreased to ~60% by day 7 [63]. In contrast, only ~20% of αβ T cells cultured from the same donor expressed CD69 [63]. Beyond acute activation, CD69 expression may have implications for tissue residency. The expression of costimulatory receptors also differed across subsets, with lower CD28 expression in γδ T cells, as previously reported [35, 64, 65]. Notably, we detected significantly lower expression of CD27, a costimulatory receptor known to play a key role in the survival and polarization of Vγ9Vδ2 T cells [66]. This CD27 deficiency may represent an opportunity for intervention aimed at enhancing CAR-T-cell survival, as discussed below.

Through comparative signalosome analysis, we identified several differentially regulated pathways. We focused on two of them for further analysis: (1) glycolysis and (2) TCR signaling. Functional metabolic profiling validated the phosphoproteomic results, confirming the prediction of a lower glycolytic rate in γδ T cells than in αβ T cells. In accordance with these observations, we found that γδ T cells expressed lower levels of GLUT-1 and exhibited lower glucose uptake. These differences were not compensated by increased oxidative phosphorylation. In fact, compared with αβ T cells, γδ T cells exhibit significantly lower oxygen consumption and lower mitochondrial mass, which we interpret as a sign of lower energy demands. A lower energy requirement has been described as an adaptation mechanism used by tumor cells, in which they “resign” functions that have a high energy cost, such as protein secretion [67]. This lower energy production may be associated with the lower secretion of cytokines observed in γδ T cells, representing an intrinsic trait of this subset. Studies on the metabolic properties of γδ T cells are extremely rare. Aehnlich et al. described Vγ9Vδ2 T cells as having “quiescent metabolism” that relies on both glycolysis and oxidative phosphorylation, with remarkable variability among donors [41]. Ishihara et al. reported that overexpression of CD8 and a TCR derived from αβ T cells induced an increase in mitochondrial activity in γδ T cells [68]. Neither of these studies involved direct comparisons between αβ and γδ T cells.

In our analysis, TXNIP emerged as a potentially actionable target on the basis of its high expression in γδ T cells. This pleiotropic protein is a well-characterized regulator of redox homeostasis, primarily through its inhibitory interaction with thioredoxin 1 (Trx-1) [69] and thioredoxin 2 (Trx-2) [70, 71] and its role as an inducer of apoptosis [72]. TXNIP upregulation occurs in exhausted T cells and quiescent naïve T cells [73], and it limits effector T-cell proliferation by binding to Trx-1 [74]. Its loss enhances IFNγ production and tumor cell death by T cells [48], and inhibiting the MondoA-TXNIP axis in a mild-acid-induced immunosuppressive environment also increases CD8+ T-cell cytotoxicity [75]. In addition, in αβ T cells, a transient decrease in TXNIP following T-cell stimulation is necessary for CD28-driven metabolic priming [76]. Mechanistically, TXNIP affects glycolysis by binding directly to the GLUT-1 receptor and inducing its internalization as well as indirectly reducing the level of GLUT-1 mRNA; ultimately, TXNIP inhibits glucose uptake [77]. TXNIP can also induce mitochondrial apoptosis in pancreatic beta cells by oxidizing Trx-2 after it is shuttled into the mitochondria during oxidative stress conditions [71]. However, to the best of our knowledge, its specific role in γδ T cells has not been described.

Building on these findings and seeking to maximize the therapeutic potential of γδ T cells for adoptive cell therapy, we designed strategies to increase Vγ9Vδ2 CAR-T-cell metabolism. We hereby provide evidence that pharmacological inhibition of TXNIP using SRI-37330 [50] during γδ ex vivo expansion increased mitochondrial mass, spare respiratory capacity, CAR expression levels, and cytokine secretion. Genomic ablation of TXNIP by CRISPR-cas9 before γδ T-cell transduction also resulted in increased CAR expression and cytokine secretion. Taken together, our results support a role for TXNIP in the metabolic phenotype of γδ T cells and warrant further exploration of the translational potential of modulating TXNIP as an enhancer of CAR-T-cell products.

In addition to TXNIP, the differential phosphorylation of transcription factors such as EOMES, HMGB2, and NFAT1 is noteworthy, as the sustained activity of these factors has been associated with T-cell exhaustion in CD8+ T cells [78–80]. Physiological expression of EOMES supports memory differentiation, cytotoxic function, and mitochondrial fitness in αβ T cells [81–83]. We postulate that these differences are associated with a cytolytic T-cell transcriptional program.

Other proteins involved in TCR signaling that are differentially phosphorylated include PLCγ1 (reduced inhibitory phosphorylation in γδ CAR-T cells) and MK14 (increased activating phosphorylation in γδ CAR-T cells). As a central mediator of early TCR signaling, PLCγ1 regulates NFAT activation and contributes to LAT-dependent signalosome formation and stability [84, 85]. Therefore, the increased PLCγ1 activity observed at 1 h in γδ CAR-T cells indicates differences in TCR signaling kinetics relative to those in αβ CAR-T cells. Notably, altered PLCγ1/LAT signaling has previously been reported between murine γδ and αβ thymocytes during development [34, 85]. Sustained activation of MK14 has been reported following stimulation of Vγ9Vδ2 T cells with either phosphoantigens or CD3 crosslinking [86]. Genetic disruption of MK14 has been associated with improved expansion, memory formation, and resistance to genomic and oxidative stress in adoptive T-cell therapy models [87], suggesting that increased MK14 activity may be detrimental to T-cell performance in the context of adoptive immunotherapy. Interestingly, MK14 can promote TXNIP expression, resulting in reduced thioredoxin activity and increased intracellular reactive oxygen species levels [88]. This mechanism may contribute to the elevated TXNIP expression we observed in γδ CAR-T cells. Direct experimental validation is needed to establish a causal relationship and to validate the role of MK14 as another actionable target to enhance the activity of γδ CAR-T cells.

Canonical TCR signaling was among the most significantly overrepresented pathways among the differentially phosphorylated proteins. Previous studies have reported a divergence between αβ and γδ TCR signaling [35, 89, 90], which has been attributed to structural variations in the components of the TCR complex, such as the composition and stoichiometry of CD3 chains [35]. This model, however, does not explain the differences that we reported because we employed the exact same CAR construct as the signal trigger in both cell subsets. Therefore, we conclude that γδ T cells present subset-intrinsic traits that condition the response to CAR-induced signaling, including decreased activation of NFAT and AP-1 (JUN) signaling. These pathways are highly relevant for T-cell proliferation and survival, and JUN signaling deficiency is associated with the induction of T-cell exhaustion [55, 56].

With that in mind, we tested the effects of supplementation of γδ CAR-T cells with a costimulatory signal designed to increase AP-1 signaling. To provide tissue specificity, we constructed a signal conditional on the presence of RANKL, a cytokine that is highly prevalent in the tumor/bone microenvironment, using a chimeric switch receptor. To avoid on-target/off-tumor toxicity, we designed our switch receptors to contain a costimulatory domain but no CD3ζ (or other ITAM-containing) domains. Therefore, RANKL binding is not expected to activate T-cell cytolytic functions on its own. Two CSR versions were generated, containing either CD27 or 41BB costimulation. While both molecules are expected to induce AP-1 signaling, CD27 was selected because of its known role in the survival of γδ T cells through the induction of antiapoptotic genes [66] and its lower expression in comparison to that in αβ T cells, as reported in this manuscript. In turn, 41BB was chosen because of its ability to increase oxidative phosphorylation in αβ CAR-T cells [91]. Eventually, we determined that the CD27-containing CSR showed superior RANKL binding and verified that it induced AP-1 signaling, preserved a central memory phenotype and promoted CAR-T-cell persistence. Further studies are needed to quantify the therapeutic benefit of this intervention in other disease settings, but our results support its application in the treatment of bone metastatic prostate cancer. Notably, our results revealed that the CSR-induced increase in AP-1 signaling occurred only in γδ T cells, further reinforcing the notion that cell product design requires tailoring to each specific T-cell subset. Importantly, our results lay the foundation for the development and translation of novel CAR-T-cell products that integrate TXNIP inhibition and/or armoring with CSRs. Current and future efforts are focused on investigational new drug (IND)-enabling studies to further characterize the pharmacological and toxicological profiles of our new products.

## Materials and methods

### Cell lines

C4-2B (CRL-3155, androgen-independent) and U266 cell lines were purchased from the American Type Culture Collection and cultured as recommended. PSCA expression was induced by transduction of C4-2B cells with a retroviral vector encoding the codon-optimized cDNA for PSCA. Deidentified healthy donor buffy coats were obtained from LifeSouth community blood centers or OneBlood (Florida Blood Services, FL). All the cell lines were periodically mycoplasma tested (MycoAlert, Lonza).

### γδ and αβ T-cell activation and expansion from PBMCs

PBMCs were isolated from buffy coats by density-gradient centrifugation. Briefly, buffy coats were diluted 1:2 in PBS (1×, pH 7.4; room temperature). In 50-mL tubes, 30 mL of diluted buffy coat was layered over 15 mL of lymphocyte separation medium. Tubes were centrifuged for 20 min at 2500 rpm and 20 °C with minimal braking. PBMC monolayers were collected, washed twice with PBS (5 min at 1500 rpm, 20 °C), resuspended in 3–4 mL of ammonium-chloride-potassium (ACK) buffer, and incubated for 10 min to lyse red blood cells. After a final PBS wash, the cells were resuspended in RPMI (5% FBS, antibiotics, 100 IU/mL IL-2). Following cell counting, γδ T cells were expanded as described [92]: PBMCs were resuspended at 1 × 10⁶ cells/mL with 4 µM Zol and plated in 24-well plates (2 mL/well); media and Zol were replaced on day 3 [93], and the cells were used for transduction on day 5. αβ T cells were expanded by resuspending PBMCs at 1 × 10⁶ cells/mL in X-Vivo (5% human serum, antibiotics, 300 IU/mL IL-2) supplemented with 5 µg/mL OKT3, after which they were plated in 24-well plates (2 mL/well) and transduced 2 days after stimulation.

### Retroviral transduction of T cells

The CAR (pMSGV1-PSCA-8t-28z or pMSGV1-BCMA-8t-28z) and CSR (pMSGV1-RANK-CD27-P2A-CD34 or pMSGV1-RANK-4-1BB-P2A-CD34) plasmids were encapsulated in an RD114-pseudotyped retrovirus generated by transient transfection of 293GP cells [94–96]. γδ and αβ T cells obtained from PBMC stimulation were transduced for 2 consecutive days in 6-well plates coated with RetroNectin® and the virus after spinoculation. For more details about the transduction protocol and quality control of the cells, please refer to the supplementary materials and methods.

Owing to the variability in γδ T-cell purity within donors, we established a threshold of >75% γδ T cells out of total CD3^+^ cells for further experiments, unless we could gate the γδ T-cell population by flow cytometry. When necessary, negative selection enrichment of γδ T cells was performed using magnetic columns and a human TCRγ/δ+ T-cellcell isolation kit, following the recommended Miltenyi protocol.

### Cytokine quantification

T-cell activity in the presence or absence of target cells was assessed by cytokine quantification. Cocultures were established in U-bottom 96-well plates with 1 × 10⁵ C4-2B PSCA⁺ tumor cells/well and a 1:1 ratio of total T cells; single cultures served as negative controls. After overnight incubation, the supernatants were collected and frozen for ELISA or ELLA analysis. ELISAs were performed using 2G1 (capture) and B133.5-biotin (detection) anti–IFN-γ antibodies, with horseradish peroxidase (HRP)-streptavidin, 3,3’,5,5’-tetramethylbenzidine (TMB), and 0.08 M H₂SO₄ for detection; the absorbance was read at 450/550 nm. ELLA of GZMB, IFN-γ, IL-2, and TNF-α was performed using precoated cartridges per the manufacturer’s instructions.

### Single-cell cytokine profiling

CAR-T-cell polyfunctionality was assessed using the Bruker Single-Cell Adaptive Immune Secretome assay. Briefly, 7 days post-transduction, αβ CAR-T cells were separated into CD4⁺ and CD8⁺ subsets and compared with enriched γδ CAR-T cells from the same donor. Cells were stimulated for 2 h with phorbol 12-myristate 13-acetate (PMA)/ionomycin or left unstimulated. After viability staining, 30,000 cells were loaded per IsoCode chip and incubated for 16 h to monitor cytokine secretion. Polyfunctionality percentages were computed using IsoLight software.

### AP-1 transcription factor activation

AP-1 activation was measured by quantifying nuclear JUN-D, c-Fos, and phospho–c-JUN levels in activated cells. A total of 5 × 10^6^ UT, CAR, or SWITCH-CAR-T cells from three donors were stimulated for 1 h at 32 °C with plate-bound hr-PSCA-Fc (0.5 μg/mL) and soluble hr-RANKL (1 μM). The cells were subsequently washed and pelleted, and nuclear proteins were extracted using an ActiveMotif Nuclear Extract kit. AP-1 complex components were quantified by colorimetry using the TransAM® AP-1 kit as described by the manufacturer.

### T-cell stimulation with plate-bound molecules

When target-cell stimulation was not feasible, T cells were activated using OKT3- or PSCA-Fc-coated plates. Coating was performed by diluting OKT3 (0.5 mg/mL) or PSCA-Fc (0.2 mg/mL) in PBS and incubating the plates for 3 h at 37 °C and 5% CO₂, followed by two PBS washes. T cells (1 × 10⁶ cells/mL in RPMI supplemented with 5% FBS, antibiotics, and 100 IU/mL IL-2) were plated at 200 μL (96-well) or 2 mL (24-well) for the indicated stimulation times.

### Phenotype characterization

Memory, costimulatory, and exhaustion markers (CD45RA, CD28, CD27, CD69, TIM-3, and PD-1; catalog numbers in the supplementary materials) were assessed on unstimulated cells by staining for 20 min at 4 °C, followed by flow cytometry on an LSRII (FACSDiva). The data were analyzed using FlowJo.

### Phosphoproteomic analysis by stable isotope labeling by amino acids in cell culture (SILAC) MS

C4-2B PSCA⁺ cells were cultured in RPMI supplemented with heavy amounts of lysine and arginine (¹³C₆¹⁴N₄-arginine, 200 mg/L; ¹³C₆-lysine, 40 mg/L) until >95% of the proteins were labeled. Twenty million tumor cells per sample were co-cultured with T cells at a 1:2 total T-cell ratio or a 2.8:1 CAR-T-cell ratio (37 °C, 5% CO₂). Six triplicate conditions were used to assess basal phosphorylation in 1) untransduced (UT) γδ or 2) UT αβ T cells after 1 h, CAR-induced basal phosphorylation in 3) γδ or 4) αβ CAR-T cells at 0 h, and antigen-induced phosphorylation in 5) γδ or 6) αβ CAR-T cells after 1 h. After incubation, the cells were washed twice with cold PBS, pelleted, and flash-frozen for protein extraction and MS  [96]. The cells were lysed, the protein concentration was measured by a Bradford assay, and aliquots of 2.8 mg (pY) and 200 mg (global phosphorylation/expression) were prepared. Proteins were reduced, cysteines alkylated, digested with trypsin, and lyophilized prior to pY enrichment.

A 24-mg pooled bulk sample was also prepared. Disulfides were reduced, cysteines alkylated, and samples were digested overnight (1:20 trypsin:substrate). Peptides were acidified with 1% trifluoroacetic acid (TFA), desalted on C18 Sep-Pak cartridges, and lyophilized. pY-enriched samples were labeled with TMT11plex; global phosphorylation samples were quenched with 5% hydroxylamine, pooled by plex, and lyophilized. Phosphopeptides were enriched by IMAC and basic reversed-phase chromatography before MS. Samples were analyzed on a nanoflow UHPLC coupled to an Orbitrap mass spectrometer, and the top 20 MS/MS spectra were acquired in a data-dependent manner (resolution 45,000; 1E5 AGC target; MaxIT 86 ms for global and 300 ms for pY; isolation window 0.8 with 0.2 offset; first mass *m/z* 100; NCE 24 and 30). A detailed protocol is provided in the supplementary materials and methods.

### Phosphoproteomic data analysis and availability

Peptides were identified using the UniProt human database. Threshold criteria are defined in more detail in the supplementary materials and methods. Reporter ion intensities were used for the relative quantification of each peptide in the TMT global pSTY data and pY data. Both data sets were normalized using IRON (iron_generic–proteomics [97]) against the 5x pooled bulk sample channels within each plex. The three injection replicates in different plex widths were averaged together to calculate _log2_ ratios between conditions by subtracting averaged sample replicates. Welch’s *t* test was used to determine the statistical significance of the difference in the log_2_ ratio. Data normalization was evaluated with scatterplots, and PCA separated samples according to the groups expected in the experimental design. The phosphoproteomic data are available from PRIDE/ProteomeXchange using the data set identifiers PXD007085 and 10.6019/PXD007085.

### Pathway enrichment analysis

Differences in phosphorylation between conditions were calculated with log_2_ ratios and *p* values obtained from Welch’s *t* test, where there were at least two replicates of phosphorylation abundance per phosphosite. The resulting log_2_ ratios, *p* values, and gene IDs per phosphosite were subsequently analyzed with Qiagen Ingenuity Pathway Analysis (IPA) software. Canonical pathway predictions were obtained with an IPA phosphorylation analysis using our own data set as a reference, setting cutoffs at a *p* value ≤ 0.05 and a log_2_ ratio ≥0.585 or ≤ -0.585.

### Western blot

T cells from three healthy donors were cocultured with C4-2B PSCA⁺ cells to induce phosphorylation of the CAR CD3ζ domain after 1, 10, 30, or 60 min of activation. Whole-cell lysates were prepared in radioimmunoprecipitation assay (RIPA) buffer supplemented with protease and phosphatase inhibitors. CAR constructs were immunoprecipitated using protein-L magnetic beads as previously described [96]. Twenty-five micrograms of protein per lane were resolved by SDS‒PAGE and transferred to nitrocellulose using the Bio-Rad Turbo protocol. The membranes were blocked in PBS + 5% BSA for 1 h at room temperature and incubated overnight at 4 °C with primary antibodies against CAR-CD3ζ and phospho-sites pY72, pY83, and pY142. Detection was performed using TidyBlot and imaged on an Odyssey Fc. Band intensities were quantified in Image Studio (v6.0) and normalized to the total CAR-CD3ζ loading control.

### SeaHorse analysis: glycolytic rate and T-cell metabolic profiling

T cells were stimulated in OKT3-coated or uncoated plates for 48 h. Seahorse assays (glycolytic rate assays or T-cell metabolic profiling kits) were performed following the manufacturer’s instructions. Briefly, the cartridges were hydrated in 200 μL/well of molecular-grade water in a 37 °C heat-only incubator for 24 h, after which the water was replaced with Seahorse XF calibrant (200 μL/well), and incubated for 1 h. In parallel, the T cells were collected, washed, and resuspended in Seahorse XF RPMI (10 mM glucose, 2 mM glutamine, and 1 mM pyruvate) at 2 × 10⁶ cells/mL. Next, 50 μL/well (1 × 10⁵ cells) was added to 96-well sample plates with ≥4 technical replicates and four background wells. The plates were subsequently centrifuged at 200 × *g* for 2 min, the degree of monolayer formation was verified, and the plates were incubated for 30 min in the same incubator.

For the glycolytic rate assays, 130 μL/well of prewarmed media was added (final volume of 180 μL/well). Rotenone/antimycin A (Rot/AA, 50 μM, 20 μL/port) and 2-deoxy-D-glucose (2-DG, 500 mM, 22 μL/port) were loaded into ports A and B, respectively. For metabolic profiling, 150 μL/well of prewarmed media was added (final volume of 200 μL/well), and ports A, B, and C were loaded with oligomycin A (13.5 μM, 25 μL/port), BAM15 (25 μM, 25 μL/port), and Rot/AA (5.5 μM, 25 μL/port). Assays were run on a Seahorse XF Pro analyzer using Wave Pro software and the manufacturer-recommended analysis templates.

### Glucose uptake

T cells were stimulated in OKT3-coated or uncoated plates for 24 h, lifted, and washed with warm PBS. The cells were resuspended in glucose-free media at 1 × 10⁶ cells/mL and incubated for 1 h at 37 °C and 5% CO₂. Glucose-Cy5 (0.2 μM) was then added, and the cells were incubated for 20 min. The cells were then washed with cold PBS; stained for CAR, CD3, TCRVδ2, and TCRαβ or TCRVδ1 for 20 min; and then gated using 4′,6-diamidino-2-phenylindole (DAPI) to determine their viability. Flow cytometry was performed immediately afterward.

### Mitochondrial mass quantification

MitoTracker Deep Red (MTDR) was reconstituted per the manufacturer’s instructions and diluted 3:1000 in prewarmed PBS + 2% BSA. T cells stimulated for 24 h (in OKT3-coated plates) or control cells were washed with warm PBS supplemented with 2% BSA and stained with antibodies against CD3, TCRαβ, TCRVδ2, CD4, CD8, and MTDR for 20 min at 37 °C. The cells were subsequently washed and resuspended in DAPI for viability before flow cytometry analysis. PBMCs were stained similarly with additional anti-CD19 and anti-TCRVδ1 antibodies. Details about the antibodies used can be found in the supplementary materials.

### Mitochondrial potential quantification

T cells were stimulated in OKT3-coated or uncoated plates for 24 h and then lifted and washed with warm (37 °C) PBS. The cells were resuspended in warm RPMI (10% FBS, antibiotics) at 1 × 10⁶ cells/mL, and each sample was split into two sets. Carbonyl cyanide m-chlorophenylhydrazone (CCCP) was added to one set (50 µM) and incubated for 15 min at 37 °C. Tetramethylrhodamine ethyl ester (TMRE, 160 nM) was then added to both sets and incubated for 30 min at 37 °C. The cells were subsequently washed with cold PBS and kept on ice for viability and surface staining. PBS-diluted yellow fixable viability dye was added for 10 min, followed by washing in cold PBS + 2% BSA and staining with antibodies against CD3, TCRαβ, and TCRVδ2 for 20 min. After the final wash, the samples were analyzed by flow cytometry. ΔMFI was calculated by subtracting each sample’s TMRE MFI from its paired CCCP-treated TMRE MFI.

### TXNIP inhibition

PBMCs were stimulated with 4 µM Zol and 5 µM SRI-37330 and plated in 24-well plates (2 × 10⁶ cells per well as described above). The media were replaced with fresh Zol and SRI-37330 on day 3. UT T cells were used on day 5 for metabolic analyses. Transductions were performed as usual, maintaining inhibitor-containing media with half-volume replacements every other day.

### TXNIP knockout

Briefly, PBMCs were expanded with Zol as described above. On day 5, γδ T cells were electroporated using an ATx electroporation system (MaxCyte, Inc.) to generate TXNIP knockout (KO) cells. Ribonucleoprotein complexes were assembled at a 2:1 molar ratio of sgRNA to Cas9 and incubated at 37 °C for 15 min. Under each condition, 20 × 10⁶ cells were resuspended in MaxCyte electroporation buffer, combined with the preformed RNP complex and electroporated using the ET-2 program. Afterward, the T cells were transferred to prewarmed media and maintained in culture for downstream analysis.

### In vivo mouse models

All procedures were approved by the University of South Florida IACUC (14112R) and followed the *Guidelines for the Care and Use of Laboratory Animals* manual published by the National Institutes of Health. Male 6-week-old NSG mice (Jackson Laboratory, #005557; Bar Harbor, ME) were intratibially injected with 5 × 10⁵ luciferase-expressing C4-2B PSCA⁺ cells [21] or 5 × 10^6^ luciferase-expressing U266 cells via the tail vein. For the prostate cancer model with C4-2B cells, 2 weeks later, the mice were randomized into four groups and treated via retro-orbital injection with PBS (untreated), untransduced (UT), CAR, or SWITCH27-CAR γδ T cells (1.2 × 10⁷ cells/mouse). Mice injected with U266 cells were randomized 3 weeks later into the same four groups, and γδ T cells (5.8 × 10^6^ cells/mouse) were administered via the tail vein. The mice received 100 IU IL-2 IP every 48 h for 2 weeks. At the endpoint, the spleen and hind limbs were collected. T cells were isolated from the spleen by mechanical dissociation and from tibial/femur bone marrow by centrifugation [98]. The cells were cryopreserved for flow cytometry. The tumor burden was monitored twice weekly by bioluminescence imaging (IVIS™ ILUMINA 200, Perkin Elmer) after D-luciferin injection. Additional details are provided in the supplementary materials and methods.

### Statistical methods

Statistical tests are specified in each figure. The phosphosite abundance data are presented as the means with individual points from three replicates run in duplicate; Welch’s *t* tests were applied when ≥2 replicates yielded values. Flow cytometry, Seahorse assays, and western blots were analyzed using paired *t* tests, with donor samples represented by distinct symbols. Significant differences (*p* ≤ 0.05) are marked with horizontal lines and asterisks. The data are shown as the means ± SDs unless otherwise indicated. Analyses were performed in GraphPad Prism 9.1.1, except for phospho-site analyses, which were conducted by the Moffitt Biostatistics and Bioinformatics Shared Resources.

## Supplementary information

Marked-up supplementary materials
