# Supplementary material for: CAR signaling instructs divergent metabolic reprogramming and functional fates in αβ and γδ T cells
Source: Cell Mol Immunol. 2026 Sep 30;23(11):1441–56. doi: 10.1038/s41423-026-01475-y (PMC13723621; doi:10.1038/s41423-026-01475-y)
Supplement: Supplementary file 1 [file 41423_2026_1475_MOESM1_ESM.docx]

**MATERIALS AND METHODS**

**T cells retroviral transduction**

γδ and αβ T cell obtained from PBMCs stimulation were transduced using RetroNectin® coated 6-well plates (20 μg/mL). Briefly, after coating the plates with 1.5 mL/well of RetroNectin® for two hours at room temperature, the wells were blocked with 2 mL of PBS + 2% BSA for 30 min at room temperature. After, wells were carefully washed with PBS; then, 2 mL of viral supernatant + 2 mL of DMEM (supplemented with 10%FBS and antibiotic) were added into each well. Spinoculation of the virus onto the wells was done by centrifuging the plates for 2 hours at 2000 g, 32 °C. Immediately after, the supernatant of each well was discarded and 4 mL of either γδ (3.5x10^6^ cells/well in RMPI) or αβ T cell (2.5x10^6^ cells/well in X-Vivo) were plated in each well. The plates were then centrifuged for 10 min at 1000 rpm, 32 °C to maximize T cell-virus interaction. A second transduction of the same cells was performed 24 hours later. Cells were kept at 37°C, 5% CO_2_ for their time in culture.

CAR expression efficiency, as well as γδ T cell content, were assessed by flow cytometry 4-6 days after transduction. The staining protocol included CAR staining either with previous incubation with biotinylated-protein L, followed by surface staining of Streptavidin-PerCP/Cy5.5, using an anti-G4S or anti-Whitlow linkers antibodies. We included antibodies to determine CD3 to gate for T cells, TCRVd2, TCRα/β to segregate αβ from γδ T cells and other markers, including a viability dye, as indicated in the pertinent figures. Flow cytometry was performed on a LSRII flow cytometer using FACSDiva software (BD Biosciences) at the Moffitt Cancer Center Flow Cytometry Core and further analyzed with FlowJo software (BD Biosciences). When necessary, the total number of CAR expressing cells was normalized by adding un-transduced cells to the sample with the highest expression of CAR until the percentages of CAR-T cells among the groups were similar.

**Phosphoproteomics analysis by stable isotope labeling by amino acids in cell culture (SILAC) mass spectrometry**

C4-2B PSCA^+^ cells were cultured in RPMI media supplemented with heavy lysine and arginine amino acids for cell labeling (^13^C_6_ ^14^N_4_ L-arginine 200 mg/L, and ^13^C_6_ L-lysine 40 mg/L) replacing the media three times per week. After four weeks, >95% of cell protein was labeled with heavy amino acids. Twenty million tumor cells per sample were co-cultured with T cells at a ratio of 1:2 total T cells, or 2.8:1 CAR-T cells (37°C, 5%CO_2_). Six different conditions were prepared by triplicates to account for: basal levels of protein phosphorylation in (1) un-transduced (UT) γδ or (2) αβ T cells after 1 hour of coculture, basal levels of CAR-induced phosphorylation in (3) γδ or (4) αβ CAR-T cells (0 hours of coculture), or antigen-CAR-recognition-induced phosphorylation events in γδ (5) or αβ (6) CAR-T cells after 1 hour of coculture. Cell mixtures were washed twice with cold PBS and centrifuged at 4 ^°^C, 300 g for 5 min post incubation times (1h, or immediately for 0h). The supernatant was discarded, and pellets were fast-frozen in dry ice for further protein extraction and MS as previously described*(1)*. Briefly, cells were lysed in denaturing buffer containing 8 M urea, 20 mM HEPES (pH 8), 1 mM sodium orthovanadate, 2.5 mM sodium pyrophosphate and 1 mM β-glycerophosphate with needle sonication, followed by centrifugation to pellet cell debris. Bradford assays determined the protein concentration for each sample. Aliquots of 2.8 mg and 200 mg were prepared for tyrosine-phosphorylation and global phosphorylation/expression, respectively. An additional pooled sample with 24 mg total protein served as a boosting sample or Bulk. Protein disulfides were reduced with 4.5 mM DTT at 60 °C for 30 minutes and then cysteines were alkylated with 10 mM iodoacetamide for 20 minutes in the dark at room temperature. Trypsin digestion was carried out at room temperature overnight with enzyme to substrate ratio of 1:20, and tryptic peptides were acidified with aqueous 1% trifluoroacetic acid (TFA) and desalted with C18 Sep-Pak cartridges according to the manufacturer’s procedure and lyophilized.

**pY Enrichment**

Following lyophilization, peptide pellets were re-dissolved in immunoaffinity purification (IAP) buffer containing 50 mM MOPS pH 7.2, 10 mM sodium phosphate and 50 mM NaCl. Phosphotyrosine-containing peptides (pY) were immunoprecipitated with p-Tyr-1000 beads (Cell Signaling Technology #8803S), according to the manufacturer’s instructions.

**Peptide labeling with Isobaric tags**

Phospho-Tyrosine (pY) enriched samples were labeled with TMT11plex and for global phosphorylation/expression, 200 mg of each tryptic digest were TMT labeled as well, following manufacture’s protocol. Label incorporation was verified to be >95% by LC-MS/MS and spectral counting (Protein Discoverer, Thermo). The samples were then quenched with aqueous 5% hydroxylamine, pooled according to each Plex and lyophilized. The TMT channel layouts were: Plex1-126C γδ CAR-T cells, 0 hour_rep1, Plex1-127N αβ CAR-T cells, 0 hour_rep1, Plex1-127C γδ CAR-T cells, 1 hour_rep1, Plex1-128N αβ CAR-T cells, 1 hour_rep1, Plex1-128C γδ CAR-T cells, 1 hour_rep2, Plex1-129N αβ CAR-T cells, 1 hour_rep2, Plex1-129C γδ un-transduced T cells, 1 hour_rep1, Plex1-130N αβ un-transduced T cells, 1 hour_rep1, Plex1-131N γδ un-transduced T cells, 1 hour_rep2, Plex1-131C bulk, and, Plex2-126 γδ CAR-T cells, 0 hour_rep2, Plex2-127N αβ CAR-T cells, 0 hour_rep2, Plex2-127C γδ CAR-T cells, 0 hour_rep3, Plex2-128N αβ CAR-T cells, 0 hour_rep3, Plex2-128C γδ CAR-T cells, 1 hour_rep3, Plex2-129N αβ CAR-T cells, 1 hour_rep3, Plex2-129C γδ un-transduced T cells, 1 hour_rep3, Plex2-130N αβ un-transduced T cells, 1 hour_rep2, Plex2-131N αβ un-transduced T cells, 1 hour_rep3, Plex2-131C bulk. Channel 130C is left empty to avoid interferences from bulk sample.

After TMT labeling, pY enriched samples were pooled into Plex1 and Plex 2, respectively and lyophilized to purify and remove non-phosphopeptides. Peptides were re-dissolved in aqueous 85% acetonitrile (ACN) containing 0.1% TFA immobilized metal affinity chromatography (IMAC) loading buffer; 20 µl of IMAC magnetic beads (Cell Signaling Technology # 20432) were washed 3x with aqueous 80% ACN, 0.1% TFA prior to incubation with immunoprecipitated peptides for 30 minutes at room temperature. Next, beads were washed 3x with aqueous 80% ACN, 0.1% TFA. Phosphopeptides were eluted twice with aqueous 50% ACN with 2.5% ammonia. Eluted peptides were dry and re-suspended in 20 µL of aqueous 2% acetonitrile/0.1% formic acid and inject 5 µL into mass spectrometer.

**Basic reversed phase liquid chromatography**

After lyophilization, for global phosphorylation samples, TMT-labeled peptides were redissolved in 250 mL of aqueous 20 mM Ammonium Formate, (pH 10.0), and subject for high pH reversed phase liquid chromatography (bRPLC) separation, performed on a XBridge 4.6 mm x 100 mm column packed with BEH C18 resin, 3.5 µm particle size, 130 Å pore size (Waters). bRPLC Solvent A was aqueous 2% ACN with 5 mM Ammonium Formate, pH 10.0. Peptides were eluted by: 5% bRPLC Solvent B (aqueous 90% acetonitrile with 5 mM Ammonium Formate, pH 10.0) for 10 minutes, 5% - 15% B in 5 minutes, 15-40% B in 47 minutes, 40-100% B in 5 minutes and 100% B held for 10 minutes, followed by re-equilibration at 1% Solvent B. The flow rate was 0.6 ml/min, and 12 concatenated fractions were collected for phosphorylation enrichment. Vacuum centrifugation (Speedvac, Thermo) dried the samples.

**IMAC enrichment**

Concatenated fractions of TMT-labeled peptides samples were re-dissolved IMAC loading buffer containing aqueous 0.1% TFA and 85% acetonitrile. Phosphopeptides were enriched using IMAC resin (Cell Signaling Technology # 20432) on a Kingfisher (Thermo). IMAC resin was washed with loading buffer. Peptides were incubated with 20 mL of resin for 30 minutes at room temperature with gentle agitation. The IMAC resin was washed twice with loading buffer followed by wash buffer (aqueous 80% ACN, 0.1% TFA). Phosphopeptides were eluted with 100 µL aqueous 50% ACN, 2.5% Ammonia. Samples were dry via vacuum centrifugation and resuspended in 20 µL of 20% acetonitrile/0.01% formic acid.

**LC-MS/MS**

A nanoflow ultra-high performance liquid chromatograph and nanoelectrospray orbitrap mass spectrometer (Dionex RSLC nano and Q Exactive HF-X, Thermo) were used for LC-MS/MS. The sample was loaded onto a pre-column (C18 PepMap100, 2 cm length x 100 µm ID packed with C18 reversed-phase resin, 5 µm particle size, 100 Å pore size) and washed for 8 minutes with aqueous 2% acetonitrile and 0.1% formic acid. Trapped peptides were eluted onto the analytical column, (C18 PepMap100, 25 cm length x 75 µm ID, 2 µm particle size, 100 Å pore size, Thermo). A 120-minute gradient was programmed as: 95% solvent A (aqueous 2% acetonitrile + 0.1% formic acid) for 8 minutes, solvent B (aqueous 90% acetonitrile + 0.1% formic acid) from 5% to 38.5% in 90 minutes, then solvent B from 50% to 90% B in 7 minutes and held at 90% for 5 minutes, followed by solvent B from 90% to 5% in 1 minute and re-equilibration for 10 minutes using a flow rate of 300 nL/min. Spray voltage was 1900 V. Capillary temperature was 275 °C. S lens RF level was set at 40. The top 20 tandem mass spectra were collected in a data-dependent manner. Settings for MS acquisition were 60,000 resolution, 3E6 AGC target, 45 ms Max IT, and recording m/z 440-2000. The settings for tandem mass spectrometry data acquisition were 45,000 resolution, 1E5 AGC target, 86 ms Max IT for global phosphorylation and 300 ms Max IT for pY samples, isolation window 0.8 with 0.2 offset, fixed first mass at m/z 100, and 2 normalized collision energy (NCE) values of 24 and 30.

**Phosphoproteomics data analysis**

MaxQuant(2) (version 1.6.14.0) was used to identify peptides using the UniProt human database (June 2020) and quantify the TMT reporter ion intensities. Up to 2 missed trypsin cleavages were allowed. The mass tolerance was 20 ppm first search and 4.5 ppm main search. Reporter ion mass tolerance was set to 0.003 Da. Carbamidomethyl cysteine was set as fixed modification. Phosphorylation on Serine/Threonine/Tyrosine and Methionine oxidation were set as variable modifications. Both peptide spectral match (PSM) and protein false discovery rate (FDR) were set at 0.05. Match between run features were activated to carry identifications across samples. Reporter ion intensities were used for the relative quantification of each peptide in the TMT global pSTY data and pY data. Both data sets were normalized using IRON (iron_generic–proteomics(3) against the 5x pooled bulk sample channels within each plex (Plex1-131C and Plex2-131C). The normalized bulk sample channels were then used to remove systematic differences between plexes. Each of these replicates was run twice. Results from both runs from the same isobaric tag in each Plex were averaged together. Then, the three injection replicates in different Plex were averaged together to calculate Log_2_ ratios between conditions by subtracting averaged sample replicates. Welch's t-test was used to determine statistical significance in the difference between the log_2_ ratio. Data normalization was evaluated with scatterplots, and PCA analysis separated samples by the groups expected on the experimental design.

**TXNIP knock out**

PBMCs from four independent healthy donors were expanded with Zol as described above. On day 5 γδ T cells were washed and centrifuged at 200g for 15 min. Ribonucleoprotein (RNP) complexes were assembled by combining TXNIP.AK (5’-TGTCAAAGCCGTTAGGATCC-3’) or non-targeting single-guide RNA (sgRNA; Integrated DNA Technologies, IDT) with Alt-R S.p. HiFi Cas9 Nuclease V3 (IDT) at a 2:1 molar ratio of sgRNA to Cas9, yielding a final Cas9 concentration of 2 µM, and incubated at 37°C for 15 min to allow RNP complex formation prior to electroporation. For each condition, 20 × 10⁶ cells were resuspended in MaxCyte electroporation buffer, combined with the pre-formed RNP complex, and transferred to an OC-100x2 processing assembly (MaxCyte). Electroporation was performed using the ET-2 program according to the manufacturer's instructions. Following electroporation, cells were rested in the processing assembly for 10 min at 37°C before transfer to pre-warmed culture medium and were maintained under standard culture conditions (37°C, 5% CO₂) for downstream analysis.

***In vivo* mouse model**

All animal experiments were performed under University of South Florida Institutional Animal Care and Use Committee (IACUC) approval (14112R). Animals were maintained on a 12-hour light/dark schedule and fed ad libitum. We tested γδ CAR-T cells in two different mouse models (male, 6-week-old, NSG mice, purchased from The Jackson Laboratory). On our castrate resistant prostate cancer, mice were intra-tibially inoculated with 20 μL of luciferase expressing C4-2B PSCA^+^ cells (5x10^5^ cells per leg diluted in PBS) as described previously(21). One day later, mice were treated with one dose of 100 ug/kg of retro-orbital Zol. Two weeks after tumor inoculation, mice were randomized into four groups to be treated with retro orbital injections of PBS (as untreated control), UT, CAR, or SWITCH27-CAR γδ T cells (1.2x10^7^ cells/mouse, in 100 μL of total volume). For the myeloma model, mice were intravenously inoculated with luciferase-expressing U266 cells (5x10^6^ cells per mouse). Three weeks later they were randomized into the four groups mentioned above and treated with 5.2x10^6^ γδ T cells via tail-vein injection. In both cases, tumor growth was monitored by measuring bioluminescence twice a week. Briefly, mice were sedated and injected intraperitoneally with 150 μg/g (body weight) of D-luciferin-potassium (GoldBio, St Louis, MO, USA), after five minutes, a ventral image of them was captured for 120 sec exposure. Mice were supplemented IP with IL-2 (100IU in 100 μL per mouse) every 48 hours for two weeks after T cell injection. At endpoint, mice were euthanized in a CO_2_ chamber, and spleen, blood, and hind limbs were collected. T cells were isolated from spleen, tibia and femur bone marrow. Briefly, spleens were mechanically dissociated, and the cells were strained through a 40µm Nailon strainer. In parallel, tibias and femurs were cut open, and the bone marrow was extracted by centrifugation. Pelleted cells were washed with PBS and then resuspended in 2mL of ACK buffer to eliminate RBCs. Finally, isolated T cells were cryopreserved for further flow cytometry analysis.

**FIGURES AND TABLES**

**Figure S1. Phenotypic differences between γδ and αβ CAR T-cells. (A)** Analysis of CAR-T cell polyfunctionality, measurement of the percentage of single cells in a T cell population able to release more than one cytokine. T cells were either resting or stimulated by PMA/Ionomycin. **(B)** Surface expression (mean ±SD) of CAR in γδ and αβ T cells. Each symbol represents T cells from an independent healthy donor (paired t-test). Representative flow cytometry histograms to the right (gated on lymphocytes/singlets/live/CD3+/ either Vδ2+ or αβTCR+ cells).

| **Protein Accession Number** | **Gene Symbol** | **Phospho sites** | **FC** | **p values** |
| --- | --- | --- | --- | --- |
| Q8TD55 | PLEKHO2 | S 389 | 3.088 | 0.043 |
| P08567 | PLEK | S 117 | 3.072 | 0.037 |
| Q49A26 | GLYR1 | S 132 | 2.451 | 0.021 |
| Q9Y2D9 | ZNF652 | T 203 | 2.444 | 0.010 |
| P16403 | H1-2 | S 36 | 2.417 | 0.009 |
| P10412 | H1-4 | S 36 | 2.339 | 0.014 |
| P20701 | ITGAL | S 1165 | 2.316 | 0.006 |
| O95936 | EOMES | S 646 | 2.294 | 0.000 |
| Q49A26 | GLYR1 | S 130 | 2.274 | 0.004 |
| Q13185 | CBX3 | S 176 | 2.267 | 0.002 |
| Q16539 | MAPK14 | Y 182 | 2.226 | 0.013 |
| Q7Z591 | AKNA | T 51 | 2.189 | 0.025 |
| Q14789 | GOLGB1 | S 1744 | 2.189 | 0.006 |
| Q14789 | GOLGB1 | S 1747 | 2.189 | 0.006 |
| Q14789 | GOLGB1 | S 1751 | 2.189 | 0.006 |
| Q8NEY8 | PPHLN1 | S 201 | 2.145 | 0.001 |
| Q8NEY8 | PPHLN1 | T 200 | 2.145 | 0.001 |
| Q86YV0 | RASAL3 | T 17 | 2.068 | 0.036 |
| Q9HCH5 | SYTL2 | S 493 | 2.047 | 0.013 |
| Q7Z591 | AKNA | S 52 | 2.007 | 0.034 |
| Q6W2J9 | BCOR | S 422 | 1.963 | 0.021 |
| Q9BPY8 | HOPX | S 70 | 1.932 | 0.045 |
| Q7Z4V5 | HDGFL2 | S 369 | 1.932 | 0.002 |
| Q7Z4V5 | HDGFL2 | S 370 | 1.932 | 0.002 |
| P23497 | SP100 | S 476 | 1.913 | 0.022 |
| O43561 | LAT | S 40 | 1.905 | 0.027 |
| O43561 | LAT | S 43 | 1.905 | 0.027 |
| Q9NRY4 | ARHGAP35 | Y 1105 | 1.904 | 0.024 |
| P22102 | GART | Y 348 | 1.888 | 0.001 |
| Q6PD62 | CTR9 | S 1072 | 1.883 | 0.003 |
| O14683 | TP53I11 | S 14 | 1.881 | 0.013 |
| Q7Z591 | AKNA | S 1228 | 1.853 | 0.005 |
| Q8N228 | SCML4 | S 277 | 1.841 | 0.000 |
| Q13469 | NFATC2 | S 53 | 1.841 | 0.020 |
| O60504 | SORBS3 | T 529 | 1.820 | 0.043 |
| Q8IVT5 | KSR1 | S 406 | 1.818 | 0.001 |
| Q8NB49 | ATP11C | S 1126 | 1.814 | 0.029 |
| P52333 | JAK3 | S 17 | 1.811 | 0.037 |
| Q9NWQ8 | PAG1 | Y 317 | 1.803 | 0.020 |
| Q8N3A8 | PARP8 | S 323 | 1.801 | 0.007 |
| Q13342 | SP140 | S 277 | 1.800 | 0.004 |
| Q9HCH5 | SYTL2 | S 562 | 1.793 | 0.026 |
| Q8NF91 | SYNE1 | T 8360 | 1.792 | 0.000 |
| Q9UQ35 | SRRM2 | S 1562 | 1.776 | 0.006 |
| Q9UQ35 | SRRM2 | S 1561 | 1.761 | 0.017 |
| Q9HCH5 | SYTL2 | S 488 | 1.759 | 0.029 |
| Q03111 | MLLT1 | S 267 | 1.755 | 0.000 |
| Q9UIK4 | DAPK2 | S 299 | 1.750 | 0.035 |
| Q9NQ75 | CASS4 | S 305 | 1.750 | 0.001 |
| Q12802 | AKAP13 | S 2500 | 1.731 | 0.023 |
| P21580 | TNFAIP3 | S 573 | 1.729 | 0.034 |
| P21580 | TNFAIP3 | S 575 | 1.729 | 0.034 |
| Q6IBW4 | NCAPH2 | S 282 | 1.699 | 0.012 |
| Q7Z591 | AKNA | S 1387 | 1.691 | 0.030 |
| Q8WVC0 | LEO1 | S 154 | 1.682 | 0.005 |
| Q99698 | LYST | S 2105 | 1.673 | 0.014 |
| Q07955 | SRSF1 | Y 189 | 1.661 | 0.049 |
| Q6IBW4 | NCAPH2 | S 284 | 1.652 | 0.043 |
| Q13469 | NFATC2 | S 73 | 1.644 | 0.032 |
| P07910 | HNRNPC | S 241 | 1.636 | 0.003 |
| O00161 | SNAP23 | S 20 | 1.632 | 0.017 |
| Q7Z6I6 | ARHGAP30 | S 709 | 1.625 | 0.027 |
| P46100 | ATRX | S 974 | 1.619 | 0.003 |
| P46100 | ATRX | T 977 | 1.619 | 0.003 |
| Q8N103 | TAGAP | S 400 | 1.606 | 0.010 |
| O14745 | SLC9A3R1 | S 280 | 1.605 | 0.004 |
| Q14005 | IL16 | S 974 | 1.604 | 0.026 |
| P17026 | ZNF22 | S 49 | 1.602 | 0.009 |
| Q9Y2D9 | ZNF652 | S 57 | 1.602 | 0.017 |
| Q9H1C0 | LPAR5 | S 320 | 1.599 | 0.021 |
| Q9NWQ8 | PAG1 | S 50 | 1.592 | 0.009 |
| Q96ST2 | IWS1 | S 54 | 1.583 | 0.001 |
| Q53EL6 | PDCD4 | S 94 | 1.572 | 0.032 |
| P49841 | GSK3B | S 219 | 1.570 | 0.016 |
| Q96JY6 | PDLIM2 | S 124 | 1.557 | 0.020 |
| Q12802 | AKAP13 | S 2498 | 1.555 | 0.016 |
| Q6PJF5 | RHBDF2 | S 385 | 1.554 | 0.004 |
| Q8IY18 | SMC5 | S 35 | 1.544 | 0.012 |
| Q8N2U9 | SLC66A2 | S 110 | 1.536 | 0.012 |
| Q7Z591 | AKNA | S 770 | 1.532 | 0.013 |
| Q96T58 | SPEN | T 1619 | 1.531 | 0.013 |
| Q92522 | H1-10 | S 33 | 1.529 | 0.006 |
| Q96T58 | SPEN | S 725 | 1.526 | 0.011 |
| P53999 | SUB1 | S 19 | 1.520 | 0.004 |
| Q5T1R4 | HIVEP3 | S 496 | 1.517 | 0.003 |
| P04150 | NR3C1 | S 134 | 1.514 | 0.009 |
| Q9Y4H4 | GPSM3 | S 35 | 1.513 | 0.000 |
| Q9Y3Q8 | TSC22D4 | S 62 | 1.510 | 0.013 |
| Q7Z4S6 | KIF21A | S 1239 | 1.506 | 0.003 |
| Q07352 | ZFP36L1 | S 54 | 1.506 | 0.002 |
| P29350 | PTPN6 | S 582 | 1.477 | 0.038 |
| P68431 | H3C13 | S 29 | 0.666 | 0.003 |
| Q6JBY9 | RCSD1 | S 82 | 0.665 | 0.003 |
| P04075 | ALDOA | S 39 | 0.664 | 0.001 |
| Q09666 | AHNAK | S 5400 | 0.664 | 0.028 |
| Q6PKG0 | LARP1 | T 526 | 0.664 | 0.000 |
| P54578 | USP14 | S 394 | 0.663 | 0.004 |
| O60841 | EIF5B | S 135 | 0.662 | 0.001 |
| O14737 | PDCD5 | S 119 | 0.662 | 0.001 |
| P22234 | PAICS | S 107 | 0.661 | 0.007 |
| Q92890 | UFD1 | S 299 | 0.661 | 0.007 |
| P84101 | SERF2 | S 21 | 0.660 | 0.005 |
| Q9NS75 | CYSLTR2 | S 102 | 0.660 | 0.037 |
| P36578 | RPL4 | S 295 | 0.660 | 0.011 |
| P78371 | CCT2 | S 260 | 0.660 | 0.032 |
| Q01826 | SATB1 | S 47 | 0.659 | 0.043 |
| P33241 | LSP1 | S 212 | 0.659 | 0.043 |
| P24941 | CDK2 | Y 15 | 0.659 | 0.001 |
| P14618 | PKM | S 37 | 0.657 | 0.003 |
| Q7Z6Z7 | HUWE1 | S 3555 | 0.656 | 0.028 |
| P21796 | VDAC1 | S 104 | 0.656 | 0.000 |
| P06748 | NPM1 | S 227 | 0.655 | 0.014 |
| Q9Y5U5 | TNFRSF18 | S 217 | 0.654 | 0.017 |
| P06748 | NPM1 | T 199 | 0.653 | 0.035 |
| Q15149 | PLEC | S 4382 | 0.653 | 0.010 |
| Q6ZSZ5 | ARHGEF18 | S 155 | 0.652 | 0.015 |
| A0FGR8 | ESYT2 | S 691 | 0.652 | 0.021 |
| A0FGR8 | ESYT2 | S 693 | 0.652 | 0.021 |
| Q8WU79 | SMAP2 | S 219 | 0.652 | 0.002 |
| P52701 | MSH6 | S 309 | 0.651 | 0.006 |
| Q9NX55 | HYPK | S 38 | 0.650 | 0.037 |
| Q99700 | ATXN2 | T 741 | 0.650 | 0.012 |
| Q09666 | AHNAK | S 4986 | 0.650 | 0.031 |
| O14578 | CIT | S 1971 | 0.649 | 0.008 |
| Q09666 | AHNAK | S 561 | 0.647 | 0.012 |
| Q15149 | PLEC | S 720 | 0.644 | 0.014 |
| P11388 | TOP2A | S 1247 | 0.643 | 0.004 |
| O43516 | WIPF1 | S 276 | 0.643 | 0.001 |
| Q15555 | MAPRE2 | S 223 | 0.642 | 0.011 |
| Q8WYL5 | SSH1 | S 576 | 0.642 | 0.004 |
| Q9ULW0 | TPX2 | S 186 | 0.640 | 0.003 |
| Q8IU68 | TMC8 | S 683 | 0.640 | 0.020 |
| Q8NFN8 | GPR156 | S 212 | 0.639 | 0.030 |
| Q8NFN8 | GPR156 | T 211 | 0.639 | 0.030 |
| Q9UPT6 | MAPK8IP3 | S 585 | 0.638 | 0.025 |
| Q01850 | CDR2 | S 145 | 0.638 | 0.043 |
| P14317 | HCLS1 | S 149 | 0.638 | 0.037 |
| Q01082 | SPTBN1 | S 2165 | 0.635 | 0.020 |
| P24941 | CDK2 CDK3 | T 14 | 0.634 | 0.003 |
| Q01082 | SPTBN1 | S 2169 | 0.630 | 0.032 |
| Q9UI08 | EVL | T 343 | 0.630 | 0.002 |
| Q14155 | ARHGEF7 | S 249 | 0.630 | 0.006 |
| Q8IWZ3 | ANKHD1 | S 1679 | 0.630 | 0.003 |
| P02794 | FTH1 | S 179 | 0.630 | 0.007 |
| Q9UQ80 | PA2G4 | S 363 | 0.626 | 0.001 |
| P21333 | FLNA | S 2152 | 0.626 | 0.002 |
| Q16566 | CAMK4 | S 360 | 0.625 | 0.006 |
| Q9BRZ2 | TRIM56 | T 442 | 0.625 | 0.001 |
| P14317 | HCLS1 | S 299 | 0.625 | 0.013 |
| Q5SW79 | CEP170 | T 969 | 0.625 | 0.040 |
| Q9H1E3 | NUCKS1 | S 181 | 0.624 | 0.010 |
| P17096 | HMGA1 | T 39 | 0.624 | 0.011 |
| Q14242 | SELPLG | S 389 | 0.623 | 0.019 |
| O43768 | ENSA | S 43 | 0.621 | 0.000 |
| P16949 | STMN1 | S 38 | 0.620 | 0.012 |
| P16150 | SPN | T 341 | 0.618 | 0.040 |
| Q6ZVF9 | GPRIN3 | S 583 | 0.616 | 0.005 |
| Q6ZVF9 | GPRIN3 | T 581 | 0.616 | 0.005 |
| P02545 | LMNA | S 423 | 0.615 | 0.002 |
| Q8N556 | AFAP1 | S 548 | 0.613 | 0.038 |
| Q8TAA9 | VANGL1 | S 42 | 0.613 | 0.039 |
| P78559 | MAP1A | S 1069 | 0.611 | 0.013 |
| P68363 | TUBA1A | S 48 | 0.611 | 0.035 |
| P19174 | PLCG1 | S 1248 | 0.610 | 0.001 |
| Q9Y3Z3 | SAMHD1 | S 23 | 0.610 | 0.044 |
| Q05655 | PRKCD | S 304 | 0.610 | 0.010 |
| P53985 | SLC16A1 | S 213 | 0.608 | 0.001 |
| Q01082 | SPTBN1 | S 2164 | 0.608 | 0.007 |
| P06733 | ENO1 | S 419 | 0.605 | 0.011 |
| Q6P6C2 | ALKBH5 | S 371 | 0.605 | 0.017 |
| Q5T4S7 | UBR4 | S 457 | 0.604 | 0.002 |
| O94804 | STK10 | S 514 | 0.604 | 0.002 |
| Q9H1E3 | NUCKS1 | T 179 | 0.603 | 0.002 |
| P27708 | CAD | S 1859 | 0.602 | 0.010 |
| O75475 | PSIP1 | T 115 | 0.599 | 0.017 |
| Q14147 | DHX34 | S 825 | 0.596 | 0.039 |
| Q14147 | DHX34 | T 831 | 0.596 | 0.039 |
| O43491 | EPB41L2 | S 598 | 0.596 | 0.017 |
| O14617 | AP3D1 | S 688 | 0.596 | 0.042 |
| Q16637 | SMN1 | S 31 | 0.595 | 0.004 |
| Q14CB8 | ARHGAP19 | S 422 | 0.593 | 0.000 |
| Q9Y2V2 | CARHSP1 | S 52 | 0.591 | 0.003 |
| Q6ZVF9 | GPRIN3 | S 734 | 0.590 | 0.000 |
| Q6ZVF9 | GPRIN3 | S 736 | 0.590 | 0.000 |
| Q01433 | AMPD2 | S 168 | 0.588 | 0.025 |
| P27361 | MAPK3 | T 202 | 0.587 | 0.007 |
| P27361 | MAPK3 | Y 204 | 0.587 | 0.007 |
| P41252 | IARS1 | S 1049 | 0.583 | 0.025 |
| Q8IY81 | FTSJ3 | S 347 | 0.583 | 0.024 |
| P23588 | EIF4B | S 283 | 0.579 | 0.047 |
| Q6JBY9 | RCSD1 | S 83 | 0.578 | 0.008 |
| P46013 | MKI67 | S 1071 | 0.573 | 0.013 |
| Q9UQC2 | GAB2 | S 405 | 0.571 | 0.016 |
| P56192 | MARS1 | S 825 | 0.570 | 0.003 |
| P24941 | CDK2 | Y 15 | 0.569 | 0.009 |
| Q16637 | SMN1 SMN2 | T 25 | 0.568 | 0.024 |
| Q00536 | CDK16 | S 153 | 0.567 | 0.032 |
| P52701 | MSH6 | S 830 | 0.566 | 0.008 |
| Q8NCD3 | HJURP | S 557 | 0.565 | 0.004 |
| P06748 | NPM1 | S 260 | 0.565 | 0.002 |
| P78559 | MAP1A | T 2105 | 0.564 | 0.004 |
| Q6ZVF9 | GPRIN3 | S 740 | 0.564 | 0.006 |
| O75534 | CSDE1 | S 514 | 0.564 | 0.012 |
| P14316 | IRF2 | S 339 | 0.563 | 0.001 |
| P51684 | CCR6 | S 347 | 0.562 | 0.048 |
| O15021 | MAST4 | S 1467 | 0.561 | 0.015 |
| P26583 | HMGB2 | S 181 | 0.561 | 0.019 |
| Q9Y570 | PPME1 | S 243 | 0.560 | 0.013 |
| P21333 | FLNA | S 1459 | 0.558 | 0.006 |
| Q13177 | PAK2 | S 141 | 0.558 | 0.016 |
| Q13303 | KCNAB2 | S 14 | 0.557 | 0.006 |
| Q9NQW6 | ANLN | S 295 | 0.556 | 0.027 |
| P09104 | ENO2 | T 265 | 0.556 | 0.001 |
| P11388 | TOP2A | S 1106 | 0.556 | 0.035 |
| Q684P5 | RAP1GAP2 | S 558 | 0.554 | 0.035 |
| P49006 | MARCKSL1 | S 104 | 0.552 | 0.014 |
| Q53QZ3 | ARHGAP15 | S 64 | 0.550 | 0.002 |
| Q9BYV9 | BACH2 | S 159 | 0.546 | 0.043 |
| P53985 | SLC16A1 | S 467 | 0.541 | 0.014 |
| Q15149 | PLEC | S 1721 | 0.539 | 0.045 |
| Q5SW79 | CEP170 | S 838 | 0.537 | 0.003 |
| Q9H019 | MTFR1L | S 238 | 0.536 | 0.005 |
| P42229 | STAT5A | Y 694 | 0.535 | 0.006 |
| P56192 | MARS1 | T 824 | 0.535 | 0.001 |
| O94851 | MICAL2 | S 515 | 0.530 | 0.007 |
| O95425 | SVIL | S 245 | 0.530 | 0.000 |
| P61978 | HNRNPK | S 214 | 0.529 | 0.000 |
| Q9UI08 | EVL | S 369 | 0.528 | 0.000 |
| Q6JBY9 | RCSD1 | S 105 | 0.525 | 0.006 |
| Q08495 | DMTN | S 85 | 0.522 | 0.034 |
| Q08495 | DMTN | S 87 | 0.522 | 0.034 |
| P11166 | SLC2A1 | S 473 | 0.521 | 0.006 |
| Q9H3M7 | TXNIP | S 361 | 0.520 | 0.036 |
| Q9Y6Q6 | TNFRSF11A | S 580 | 0.519 | 0.026 |
| Q9Y4F9 | RIPOR2 | S 37 | 0.519 | 0.031 |
| P06733 | ENO1 | Y 44 | 0.519 | 0.034 |
| P30050 | RPL12 | S 38 | 0.518 | 0.001 |
| P05771 | PRKCB | T 642 | 0.517 | 0.040 |
| P22314 | UBA1 | S 835 | 0.516 | 0.003 |
| Q9NSI8 | SAMSN1 | S 74 | 0.515 | 0.006 |
| Q5SW79 | CEP170 | S 971 | 0.515 | 0.008 |
| Q13415 | ORC1 | S 311 | 0.513 | 0.000 |
| Q96BY6 | DOCK10 | S 12 | 0.507 | 0.017 |
| P60174 | TPI1 | S 21 | 0.504 | 0.000 |
| O75569 | PRKRA | S 18 | 0.502 | 0.000 |
| P16949 | STMN1 | S 25 | 0.499 | 0.000 |
| P62753 | RPS6 | S 247 | 0.499 | 0.022 |
| Q6JBY9 | RCSD1 | S 108 | 0.495 | 0.006 |
| Q96TA1 | NIBAN2 | S 665 | 0.491 | 0.004 |
| Q9Y3Z3 | SAMHD1 | T 592 | 0.490 | 0.000 |
| P49795 | RGS19 | S 65 | 0.490 | 0.001 |
| O43491 | EPB41L2 | S 87 | 0.486 | 0.004 |
| P62753 | RPS6 | S 240 | 0.485 | 0.000 |
| Q9Y5K6 | CD2AP | S 224 | 0.483 | 0.001 |
| Q9UI08 | EVL | S 283 | 0.483 | 0.021 |
| P27708 | CAD | S 1407 | 0.482 | 0.029 |
| Q8IY18 | SMC5 | S 131 | 0.477 | 0.007 |
| Q8IY18 | SMC5 | T 137 | 0.477 | 0.007 |
| A0JNW5 | UHRF1BP1L | S 953 | 0.477 | 0.009 |
| P62753 | RPS6 | S 235 | 0.474 | 0.004 |
| P41236 | PPP1R2 | S 20 | 0.474 | 0.001 |
| Q12965 | MYO1E | S 1002 | 0.473 | 0.003 |
| Q13177 | PAK2 | S 64 | 0.463 | 0.008 |
| B0I1T2 | MYO1G | S 1010 | 0.462 | 0.013 |
| P62753 | RPS6 | T 241 | 0.460 | 0.003 |
| Q9H3M7 | TXNIP | T 349 | 0.454 | 0.018 |
| Q9H3M7 | TXNIP | T 348 | 0.450 | 0.006 |
| P62857 | RPS28 | S 23 | 0.450 | 0.017 |
| Q96TA1 | NIBAN2 | S 692 | 0.449 | 0.001 |
| Q96TA1 | NIBAN2 | S 696 | 0.448 | 0.001 |
| P48167 | GLRB | S 411 | 0.441 | 0.040 |
| P48167 | GLRB | T 410 | 0.441 | 0.040 |
| Q14242 | SELPLG | S 358 | 0.441 | 0.002 |
| P62753 | RPS6 | S 236 | 0.440 | 0.003 |
| Q9UIQ6 | LNPEP | S 80 | 0.428 | 0.000 |
| Q86VZ1 | P2RY8 | S 324 | 0.422 | 0.003 |
| P31350 | RRM2 | S 20 | 0.414 | 0.001 |
| P35611 | ADD1 | S 726 | 0.410 | 0.003 |
| P51692 | STAT5B | Y 699 | 0.405 | 0.003 |
| Q9H3M7 | TXNIP | S 346 | 0.400 | 0.015 |
| Q96TA1 | NIBAN2 | S 691 | 0.400 | 0.011 |
| Q6ZVF9 | GPRIN3 | S 462 | 0.397 | 0.002 |
| Q09666 | AHNAK | S 5830 | 0.396 | 0.000 |
| P33993 | MCM7 | Y 600 | 0.392 | 0.014 |
| P05412 | JUN | S 58 | 0.388 | 0.001 |
| O00148 | DDX39A | S 183 | 0.386 | 0.000 |
| P22234 | PAICS | S 27 | 0.385 | 0.008 |
| P41236 | PPP1R2 | T 21 | 0.374 | 0.001 |
| P13612 | ITGA4 | S 1021 | 0.366 | 0.000 |
| Q9Y4F9 | RIPOR2 | S 21 | 0.364 | 0.000 |
| P16949 | STMN1 | S 16 | 0.351 | 0.000 |
| P00558 | PGK1 | S 203 | 0.342 | 0.000 |
| P11171 | EPB41 | S 85 | 0.338 | 0.013 |
| Q5SW79 | CEP170 | S 446 | 0.337 | 0.000 |
| Q5SW79 | CEP170 | S 450 | 0.336 | 0.042 |
| P78559 | MAP1A | S 2629 | 0.331 | 0.002 |
| Q13642 | FHL1 | S 98 | 0.327 | 0.000 |
| P78559 | MAP1A | S 2617 | 0.321 | 0.007 |
| P09972 | ALDOA | Y 204 | 0.316 | 0.009 |
| P13598 | ICAM2 | T 259 | 0.302 | 0.001 |
| Q9H400 | LIME1 | S 70 | 0.273 | 0.001 |
| P78559 | MAP1A | S 986 | 0.271 | 0.029 |
| Q6UX15 | LAYN | S 299 | 0.258 | 0.011 |
| P01730 | CD4 | S 440 | 0.248 | 0.014 |
| Q9UN19 | DAPP1 | Y 139 | 0.236 | 0.008 |
| P01732 | CD8A | S 229 | 0.212 | 0.001 |
| P01732 | CD8A | S 231 | 0.183 | 0.000 |

**Table S1. Differentially phosphorylated proteins sites between activated γδ and αβ CAR T cells.** S: Serine, T: Threonine, Y: Tyrosine.

**Figure S2. Phosphoproteomics approach to determine CAR-driven differential signaling.** Diagram of the experimental design to analyze phosphorylation events triggered by CAR activation. Briefly, T cells were expanded from healthy donor PBMCs with either Zoledronate (Zol) or OKT3. Once activated, T cells were transduced with a retroviral vector to express PSCA-8t28z CAR. Five days later, CAR expression was confirmed by flow cytometry. In parallel, PSCA-expressing tumor cells (C4-2B) were cultured with SILAC media until >95% of their proteome was labelled with ^13^C_6_ ^14^N_4_ L-arginine, and ^13^C_6_ L-lysine. T cells and tumor cells are co-cultured for either 1h at 37°C or “0h” and pelleted down at 4°C. Pellets are washed with cold PBS and quickly frozen for further analysis. After protein extraction and digestion, phosphorylated peptides are enriched and analyzed by tandem mass spectrometry. Heavy isotope-labeled peptides from tumor cells were filtered out, and unlabeled peptides were mapped to the proteome and quantified. Finally, differentially phosphorylated proteins were identified as those having a fold change greater than 1.5 and a p-value (Welch’s t-test) of less than 0.05.

**Figure S3. Metabolic differences between γδ and αβ CAR-T cells. (A)** Bar chart of the relative abundance (normalized by αβ unstimulated UTs, represented by the dotted red line) of glycolytic enzymes in unstimulated and 24h-OKT3 stimulated T cells (mean ±SD; n=3). **(B)** Bar chart of surface expression of glucose receptor GLUT-1 in 24h-OKT3 stimulated T cells (mean ± SD; n=8). **(C)** Bar chart of surface expression of alternative glucose receptors GLUT-3, GLUT-4, GLUT-6, and GLUT-8 in non-cultured PBMCs (mean ± SD; n=9). **(D)** Proton efflux rate (PER) of 48h-OKT3 stimulated T cells. Representative donor. Rot/AA: rotenone and antimycin A, 2DG: 2-Deoxy-D-glucose. **(E)** Bar charts representing the Glycolytic Proton Efflux Rate (GlycoPER) as an indirect measurement of basal glycolysis and compensatory glycolysis levels of 48h-OKT3 stimulated T cells (mean ±SD; n=12). **(F)** Oxygen consumption rate (OCR) of 48h-OKT3 T cells. Representative donor. Oli: Oligomycin, BAM15: C16H10F2N6O. **(G)** Bar charts of the OCR as indirect measurement of basal respiration, maximal respiration and spare respiratory capacity (SRC) of 48h-OKT3 stimulated T cells (mean ±SD; n=6). **(H)** Bar chart of MitoTracker™ DeepRed (MTDR) geometric mean fluorescence intensity (gMFI) on 24h-OKT3 stimulated T cells (mean ±SD; n=8). **(I)** Bar chart of the delta Tetramethylrhodamine (TMRE) MFI as an indirect measurement of the mitochondrial membrane potential of 24h-OKT3 stimulated T cells (mean ±SD; n=5). The data points in (B) were collected from lymphocytes/singlets/live/CD3+/ either Vδ2+ or αβTCR+ gates; in (C) were collected from lymphocytes/singlets/live/CD45+/either CD19+, CD3+/αβTCR+, CD3+/ Vδ1+, or CD3+/ Vδ2+ gates. The symbols from each bar in (B, C, E, G-I), represent an independent healthy donor sample between and within experiments. Statistically significant differences in (B, C, E, G-I) are annotated as * for p < 0.05, ** for p < 0.01, *** for p < 0.001, and **** for p < 0.0001 (paired t-test).

**Figure S4. Original TXNIP Western Blot Images.** TXNIP expression from four independent donors (HD) whole protein lysate. Samples are γδ CAR-T cells in different culture conditions including CRISPRcas9 cells TXNIP KO or non-targeting control (NTC). A representative sample is shown in Figure 4M. TXNIP expression quantification was normalized by β-actin.

**Figure S5. Original CD3ζ Western Blot Images.** Images from the immunoprecipitated CAR proteins of three healthy donors after T cells were cocultured with C4-2B PSCA^+^ cells for a time course of 0, 1, 10, 30 or 60 min. Intensity of CD3z total (first column to the left) was used to normalize phospho-sites (pY72, pY83, or pY142) from the same run. One representative sample was selected to illustrate Figure 5D in the main manuscript.

**Figure S6. Effects of SWITCH27 in αβ CAR-T cells and in anti-BCMA γδ CAR-T cells. (A)** Flow cytometry analysis of the proportion of CAR+/CD34+ αβ T cells (mean ± SD, n = 5, independent healthy donors). **(B)** Relative abundance of AP-1 proteins in the nuclei of αβ T cells analyzed by TransAM AP-1 activation assay (ActiveMotif, mean ± SD, n = 3, independent health donors). **(C)** Matrix of statistical comparisons of average radiance per day from the tumor growth curve shown in **Figure 6I**. Significant differences are highlighted in red. **(D)** Mean bioluminescence of tumors (photons×seconds^−1^×centimeter^−2^×steradian^−1^) from untreated, γδ UT, γδ CAR, or γδ SWITCH27-CAR-Treated mice (n = 5 mice/group). Representative images shown below. γδ CAR-T cells were infused on day 0 (arrow). Statistical significance shown to the right. **(E)** Matrix of statistical comparisons of average radiance per day from the tumor growth curve shown in panel (D). Significant differences are highlighted in red. (F) γδ T cell count from spleen and bone marrow (femur and tibia) of treated mice. Data from (A) reflects lymphoid/singlet/live/CD3+/abTCR+ cells, representative flow cytometry plots are shown. * p < 0.05, ** p < 0.01, (B) paired t-test; (C, E) Welsh t-test; (D) Welsh t-test comparing CAR (purple) or SWITCH27-CAR (green) against the untreated control; (F) unpaired t-test.

| **Reagent name and specifications** | **Catalog #** | **Company** |
| --- | --- | --- |
| PBS (pH7.4) | 10-010-049 | GIBCO |
| Ammonium-Chloride-Potassium (ACK) Lysing buffer | A10492-01 | GIBCO |
| RPMI 1640 with GlutaMAX | 61-870-127 | GIBCO |
| DMEM | 11965-118 | GIBCO |
| Antibiotic-Antimycotic 100X | 15240-062 | GIBCO |
| MycoAlert, mycoplasma test | LT07-318 | Lonza |
| D-luciferin-potassium salt | LUCK-1G | GoldBio |
| TCRγ/δ+ T Cell Isolation Kit, human | 130-092-892 | Miltenyi Biotec |
| Zoledronic Acid (Zol) | S1314 | Selleckchem |
| Lymphocyte Separation Media (LSM) | 850494 | MP Biomedicals |
| RPMI 1640 for SILAC media | 88365 | ThermoFisher Scientific |
| IFN-γ monoclonal antibody 2G1 | M700A | ThermoFisher Scientific |
| IFN-γ monoclonal biotinylated antibody B133.5 | M701B | ThermoFisher Scientific |
| HRP-conjugated streptavidin | N100 | ThermoFisher Scientific |
| 3,3′,5,5′-Tetramethylbenzidine | 34028 | ThermoFisher Scientific |
| LIVE/DEAD™ Fixable Near-IR Dead Cell, for 633 or 635 nm | L10119 | ThermoFisher Scientific |
| LIVE/DEAD™ Fixable Yellow Dead Cell, for 405 nm | L34968 | ThermoFisher Scientific |
| CellTrace™ Violet Cell Proliferation kit | C34571 | ThermoFisher Scientific |
| Lipofectamine® 2000 | 11668500 | ThermoFisher Scientific |
| Biotin-Protein L | M00097 | GenScript |
| Proleukin (IL-2) | NDC 76310-022-01 | Prometheus Laboratories |
| 13C6 14N4 L-arginine | 608033 | Sigma-Aldrich |
| 13C6 L-lysine | 643459 | Sigma-Aldrich |
| Phosphatase inhibitors | P5726-1ML, P0044-1ML | Sigma-Aldrich |
| Bovine Serum Albumin (BSA) | A3803-100G | Sigma-Aldrich |
| GlucoseCy5 | SML3233 | Sigma-Aldrich |
| X-Vivo™ 15 | 04-418Q | Lonza |
| Human serum AB (HSAB) | 100-512-100 | Gemini-bioproducts |
| Heat-inactivated Fetal Bovine Serum (FBS) | A021011 | Gemini-bioproducts |
| RetroNectin® | T100B | Takara Bio |
| Human PSCA Protein, ECD (Extracellular Domain), Fc-fusion | FCL2506 | G&P Biosciences |
| Sea-Horse XF RPMI and supplements | 103576-100 | Agilent |
| Sea-Horse XF calibrant, plates and sensor cartridges (XFe96 FluxPak mini) | 102601-100 | Agilent |
| Sea-Horse Glycolytic Assay Rate kit | 103710-100 | Agilent |
| Sea-Horse T Cell Metabolic Profiling kit | 103772-100 | Agilent |
| 2-(N-(7-Nitrobenz-2-oxa-1,3-diazol-4-yl)Amino)-2-Deoxyglucose (2-NBDG) | N13195 | Invitrogen |
| Mitotracker Deep Red™ | M46753 | Invitrogen |
| Pierce™ Protein L Magnetic Beads | 88849 | Invitrogen |
| Protease inhibitors | 4693116001 | Roche |
| Trans-Blot Turbo RTA Midi 0.2 µm Nitrocellulose Transfer Kit | 1704271 | Bio-Rad |
| 4–20% Mini-PROTEAN® TGX™ Precast Protein Gels, 15-well | 4561096 | Bio-Rad |
| Precision Plus Protein™ ladder All Blue | 1610373 | Bio-Rad |
| TidyBlot Western Blot Detection Reagent | STAR209PA | Bio-Rad |
| Mitochondrial Membrane Potential Assay kit (II) (TMRE, CCCP and PBS) | 13296 | Cell Signaling Technology |
| G4S Linker (E7O2V) Rabbit Monoclonal Antibody (PE Conjugate) | 38907 | Cell Signaling Technology |
| Whitlow/218 Linker (E3U7Q) Rabbit Monoclonal Antibody (Alexa Fluor® 488 Conjugate) | 55809 | Cell Signaling Technology |
| p-Tyr-1000 beads | 8803S | Cell Signaling Technology |
| IMAC magnetic beads | 20432 | Cell Signaling Technology |
| IMAC resin | 20432 | Cell Signaling Technology |
| Customized ELLA simple plex (granzyme B, IFN-γ, IL-2 and TNF-α) cartridge kits | NA | Biotechne® / Protein Simple |
| Nuclear Extract Kit | 40010 | ActiveMotif |
| TransAM ® AP-1 c-Fos | 44096 | ActiveMotif |
| TransAM ® JUND | 43496 | ActiveMotif |
| TransAM ® c-Jun | 46096 | ActiveMotif |
| E-Plates 96 PET | 300600900 | ACEA Biosciences |
| Anti-human CD45-BV785 (Clone HI30) | 304048 | BioLegend |
| Anti-human CD3-BV711 (clone OKT3) | 317328 | BioLegend |
| DAPI dye | 422801 | BioLegend |
| Anti-human CD4-PECy7 (clone RPA-T4) | 317442 | BioLegend |
| Anti-human CD19-BV605 (clone HIB-19) | 302243 | BioLegend |
| Anti-human CD314-BV785 (NKG2D, clone 1D11) | 320829 | BioLegend |
| Anti-human PD-1-AF700 (clone EH12.2H7) | 329952 | BioLegend |
| Anti-human CD28-PerCPCy5.5 (clone CD28.2) | 302922 | BioLegend |
| Anti-human CD27-BV605 (clone O323) | 302830 | BioLegend |
| Streptavidin-BV421 | 563259 | BioLegend |
| Anti-human TCRVd2-PE (clone 123R3) | 130-125-855 | Miltenyi Biotec |
| Anti-human TCRVd2-APC (clone 123R3) | 130-121-339 | Miltenyi Biotec |
| Anti-human TCRVd1-FITC (clone REA173) | 130-118-362 | Miltenyi Biotec |
| Anti-human TCRVd1-VioGreen (clone REA173) | 130-120-442 | Miltenyi Biotec |
| Anti-human TCRα/β-APC (clone REA652) | 130-113-535 | Miltenyi Biotec |
| Anti-human TCR αβ-BV786 (clone T10B9.1A-31) | 563825 | BD biosciences |
| Anti-human CD3-BUV395 (clone SK7) | 564001 | BD biosciences |
| Anti-human CD8-BUV395 (clone RPA-T8) | 563795 | BD biosciences |
| Anti-human CD8-PE-Cy7 (clone RPA-T8) | 557746 | BD biosciences |
| Anti-human GLUT1-AF647 (clone 202915) | 566580 | BD biosciences |
| Anti-human TIM3-BV650 (clone 7D3) | 565564 | BD biosciences |
| Anti-human CD69-BV650 (clone FN50) | 563835 | BD biosciences |
| Anti-human CD25-BV711 (clone 2A3) | 563159 | BD biosciences |
| Anti-human phosphoY142 CD3z (clone K25-407.69) | 558402 | BD biosciences |
| Anti-human GLUT4-PECy7 (polyclonal) | NBP1-49533PECY7 | Novus Biologicals |
| Anti-human GLUT6-AF405 (polyclonal) | NBP1-59891AF405 | Novus Biologicals |
| Anti-human GLUT3-PECy7 (clone 202017) | FAB1415G-100UG | R&D Systems |
| Anti-human GLUT8-PE (polyclonal) | orb2574936 | Biorbyt |
| Anti-human CD3z (clone F-3) | sc-166275 | Santa Cruz |
| Anti-human phospho-Y83 CD3z (clone EP776(2)Y) | ab303153 | Abcam |
| Anti-human phospho-Y72 CD3z (polyclonal) | SAB4200330 | Millipore Sigma |
| Single-Cell Adaptive Immune Chip – (H) 8 | ISOCODE-1001-8 | Bruker Cellular Analysis |
| OC-100x2 processing assembly | SOC-1x2 | MaxCyte |
| Electroporation buffer | EPB-1 | MaxCyte |
| Alt-R™ S.p. HiFi Cas9 Nuclease V3 | 1081060 | IDT |
| Alt-R™ CRISPR-Cas9 sgRNA | Custom | IDT |
| Cas9 Negative Control sgRNA #1 | Custom | IDT |
| anti-TXNIP Rabbit monoclonal antibody clone (D5F3E) | 14715S | Cell Signaling |

**Table S2. Reagents.**

**REFERENCES**

1.	Ramello MC, Benzaid I, Kuenzi BM, Lienlaf-Moreno M, Kandell WM, Santiago DN, et al. An immunoproteomic approach to characterize the car interactome and signalosome. Science signaling. 2019;12:eaap9777.

2.	Cox J, Mann M. Maxquant enables high peptide identification rates, individualized p.P.B.-range mass accuracies and proteome-wide protein quantification. Nat Biotechnol. 2008;26:1367-1372.

3.	Welsh EA, Eschrich SA, Berglund AE, Fenstermacher DA. Iterative rank-order normalization of gene expression microarray data. BMC Bioinformatics. 2013;14:153.
